# Discovery of an orally active benzoxaborole prodrug effective in the treatment of Chagas disease in non-human primates

**DOI:** 10.1038/s41564-022-01211-y

**Published:** 2022-09-05

**Authors:** Angel M. Padilla, Wei Wang, Tsutomu Akama, David S. Carter, Eric Easom, Yvonne Freund, Jason S. Halladay, Yang Liu, Sarah A. Hamer, Carolyn L. Hodo, Gregory K. Wilkerson, Dylan Orr, Brooke White, Arlene George, Huifeng Shen, Yiru Jin, Michael Zhuo Wang, Susanna Tse, Robert T. Jacobs, Rick L. Tarleton

**Affiliations:** 1 Center for Tropical and Emerging Global Diseases and Department of Cellular Biology, University of Georgia, Athens, GA USA; 2 Anacor Pharmaceuticals, Inc, Palo Alto, CA USA; 3 College of Veterinary Medicine and Biomedical Sciences, Texas A&M University, College Station, TX USA; 4 Michale E. Keeling Center for Comparative Medicine and Research of The University of Texas MD Anderson Cancer Center, Bastrop, TX USA; 5 Department of Pharmaceutical Chemistry, The University of Kansas, Lawrence, KS USA; 6 Pharmacokinetics, Dynamics and Metabolism, Worldwide Research, Pfizer Inc., New York, NY USA

**Keywords:** Target identification, Parasitic infection, Parasite biology, Drug development

## Abstract

*Trypanosoma cruzi*, the agent of Chagas disease, probably infects tens of millions of people, primarily in Latin America, causing morbidity and mortality. The options for treatment and prevention of Chagas disease are limited and underutilized. Here we describe the discovery of a series of benzoxaborole compounds with nanomolar activity against extra- and intracellular stages of *T. cruzi*. Leveraging both ongoing drug discovery efforts in related kinetoplastids, and the exceptional models for rapid drug screening and optimization in *T. cruzi*, we have identified the prodrug AN15368 that is activated by parasite carboxypeptidases to yield a compound that targets the messenger RNA processing pathway in *T. cruzi*. AN15368 was found to be active in vitro and in vivo against a range of genetically distinct *T. cruzi* lineages and was uniformly curative in non-human primates (NHPs) with long-term naturally acquired infections. Treatment in NHPs also revealed no detectable acute toxicity or long-term health or reproductive impact. Thus, AN15368 is an extensively validated and apparently safe, clinically ready candidate with promising potential for prevention and treatment of Chagas disease.

## Abstract (web summary)

A newly discovered benzoxaborole prodrug AN15368 cures *Trypanosoma cruzi* infection (the cause of Chagas disease) in mice and in naturally infected non-human primates.

## Main

Chagas disease, caused by the protozoan parasite *Trypanosoma cruzi*, remains the highest-impact parasitic disease in Latin America and one of the major causes of infection-induced myocarditis worldwide^1^. For more than five decades, two nitroheterocyclic compounds, benznidazole and nifurtimox, have been available for treatment of the infection, but are relatively rarely used due to their inconsistent efficacy and high frequency of side effects. Recent trials of potential new therapies have yielded disappointing results^2,3^.

Among the challenges for drug development in *T. cruzi* infection is the parasite’s predominantly intracytoplasmic location in mammals and its ability to invade a wide variety of host cell types and tissues, although it shows a clear preference for muscle cells, including cardiac, skeletal and smooth muscle of the gut. The recent discovery of arrested ‘dormant’ intracellular forms of *T. cruzi* that are relatively and transiently resistant to otherwise highly effective trypanocidal compounds^4^ may partially explain why these therapeutics must be given for extended periods of time (60 d is common) but still have a high failure rate.

Previous work from our laboratories have identified a novel class of boron-containing molecules, the benzoxaboroles^5,6^ as having potent activity against protozoans including *Trypanosoma brucei*^7^*, Leishmania donovani*^8^ and *Plasmodium falciparum*^9^. Screening of the Anacor benzoxaborole compound library against *T. cruzi* revealed several hits, but initial assessment of structure-activity relationships (SARs) suggested limited opportunity for improvement of potency and/or selectivity, particularly in those subclasses previously found to have activity against *T. brucei* and *L. donovani*. Here we have taken advantage of this benzoxaborole scaffold and the multiple natural host species for *T. cruzi* to move rapidly from in vitro detection of trypanocidal activity in lead compounds into facile in vivo tests of efficacy in mice and ultimately in naturally infected non-human primates (NHPs). The result is identification of a class of benzoxaboroles that provide high rates of parasitological cure of *T. cruzi* infection. AN15368 from this class is the first, extensively validated and safe potential clinical candidate in over 50 yr for the prevention/treatment of Chagas disease.

## Results

### In vitro activity and SARs

**Fig. 1:**
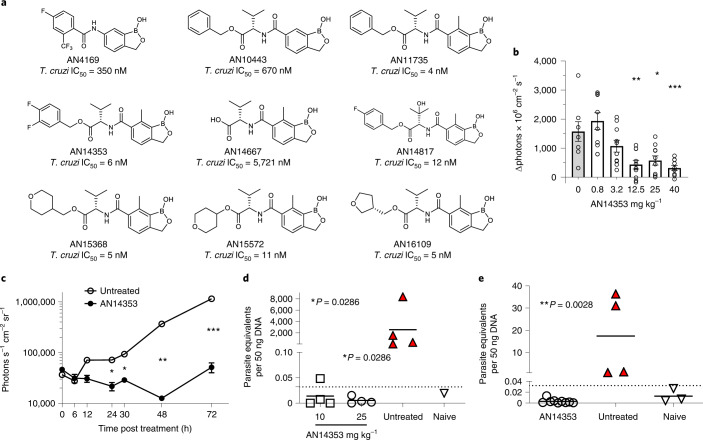
Selected benzoxaboroles are active against *T. cruzi* in vitro and in vivo. **a**, Structure of key compounds evaluated, including the ultimate clinical candidate, AN15368. **b**, C57BL/6J mice received a single oral dose of AN14353 at the indicated concentration at 2 dpi with 2.5 × 10^5^ tdTomato-expressing *T. cruzi* infection in the footpads. The relative increase in the fluorescent intensity of the footpads between 2 and 4 dpi was assessed by in vivo imaging (untreated: *n* = 8; AN15368: *n* = 10 individual feet). Asterisks represent the statistical significance of the difference between each treated group and the untreated control. **c**, C57BL/6J mice infected in the footpads with 2.5 × 10^5^ luciferase-expressing parasites received a single oral dose of AN14353 (25 mg kg^−1^) or no treatment at 3 dpi and the bioluminescence signal of the feet was measured over 72 h (untreated: *n* = 2; AN15368: *n* = 6 individual feet). **d**, Daily oral treatment with AN14353 at 10 or 25 mg kg^−1^ was administered for 40 consecutive days to C57BL/6J mice starting at 15 d post intraperitoneal infection with 10^4^ *T. cruzi* trypomastigotes. Parasite load in skeletal muscle was determined by qPCR following cyclophosphamide-induced immunosuppression (untreated and treated groups: *n* = 4 mice; naïve *n* = 1). **e**, IFN-gamma deficient mice intraperitoneally infected with *T. cruzi* received a daily oral treatment with AN14353 (25 mg kg^−1^) for 40 d (AN15368: *n* = 4; untreated: *n* = 9 mice; naïve *n* = 3). Parasite load in skeletal muscle after immunosuppression with cyclophosphamide was assessed by qPCR. Untreated animals in **e** were killed and tissue samples collected before the end of the experiment due to the high susceptibility to *T. cruzi* infection and the lethal pathology developed by IFN-gamma-deficient animals. Red filled symbols denote animals in which parasites were detected by fresh blood smears or haemoculture. Dotted lines represent the cut-off in the qPCR assays. Data are presented as mean ± s.e.m. For **b**, **d** and **e**: Mann-Whitney test, two tailed; for **c**: unpaired *t*-test, 95% confidence interval. *P ≤ 0.05; **P ≤ 0.01; ***P ≤ 0.001. Source data

**Extended Data Fig. 1:**
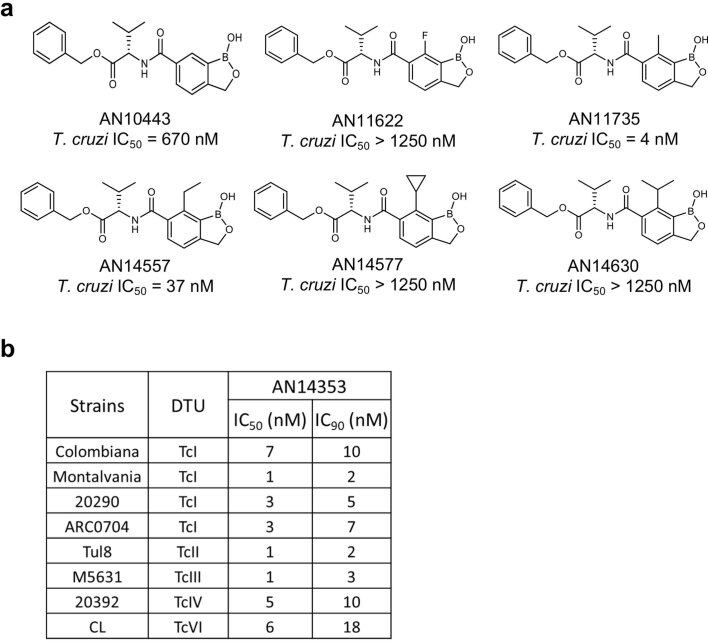
Structure activity relationships of benzoxaboroles and their activity on different genetic types of *T. cruzi*. **a**. Substitutions in the benzoxaborole ring C(7) position of AN10443 strongly affects in vitro activity against *T. cruzi*, with larger substitutions decreasing activity. **b**. *T. cruzi* of diverse genetic types (DTUs) are susceptible to AN14353-mediated killing (n = 3 biological replicates for each profile determination).

The initial lead benzoxaborole 6-carboxamide AN4169 (Fig. 1) provided 100% cure of mice infected with the *T. cruzi* Brazil strain^5^; however, rodent tolerability studies suggested that an insufficient therapeutic margin existed for further progression of this compound. Profiting from a concurrent project evaluating analogues of AN4169 against *Trypanosoma congolense*^10^, several compounds with submicromolar activity against *T. cruzi* in vitro and good metabolic stability in an in vitro mouse S9 liver fraction assay were identified, among these an ester of the 6-valine ‘transposed’ carboxamide AN10443 (Fig. 1). Further manipulation of this compound, in particular inclusion of a methyl group at C(7) of the benzoxaborole ring as in AN11735, drastically increased in vitro activity against *T. cruzi* (IC_50_ < 10 nM), whereas substituents larger than methyl at C(7) ablated activity (Extended Data Fig. 1a).

SAR development of the ester region in the C(7)-methyl series showed that potency was not substantially impacted by substituents on the benzyl ester, with the exception of the 4-(3-pyrrolidinylethyloxy) analogue AN14502 and the 4-methylsulfonyl analogue AN14561, which were markedly less potent (Supplementary Table 1). Physicochemical properties were more affected, with most simple halogenated analogues being poorly soluble in aqueous media. Metabolic stability, as estimated by incubation with the mouse S9 liver fraction, was variable, and roughly tracked with lipophilicity (cLogD; Supplementary Table 1).

These observations prompted us to more substantially modify the ester region of the molecule through preparation of aliphatic and heterocyclic esters that would be expected to be less lipophilic, more water soluble and less susceptible to metabolism (Supplementary Table 2). Several interesting SARs emerged from this group of analogues: (1) esters containing basic amines (for example, AN15143, AN15144, AN15658, AN15678, AN15129, AN15192, AN15078, AN14504 and AN15159) were less active than neutral compounds and (2) small aliphatic esters were quite potent except for the t-butyl ester (AN15134). The relationship between lipophilicity and solubility or metabolic stability continued to exist for these compounds and provided reasonably wide latitude for modulation of such properties by choice of ester substituent.

### In vivo activities

In addition to being very potent in vitro, the valine esters were also of generally good stability, including in mouse and human S9 liver fraction assays (Supplementary Table 3). Several valine esters also exhibited low clearance (<20% hepatic blood flow) following intravenous dosing and good bioavailability following oral administration to mice, achieving good to excellent exposure (area-under-the-curve (AUC) > 10 µg h ml^−1^) with low mg kg^−1^ doses (Supplementary Table 3). Concurrent testing results of these compounds in vivo for the ability of a single oral dose to reduce an established focal infection in the footpad of mice over 3 d^11,12^ were very encouraging, with AN14353 emerging as the lead on the basis of activity at reduced doses (Fig. 1b). Notably, AN14353 demonstrated rapid in vivo trypanocidal activity (Fig. 1c), had high in vitro potency for a range of *T. cruzi* isolates for different genetic lineages (discrete typing units, DTUs; Extended Data Fig. 1b) and could consistently resolve established *T. cruzi* infections at a dose of 25 mg kg^−1^ in a standard^5,13^ 40 d treatment protocol in wild-type mice (Fig. 1d) as well as infections in immunodeficient mice (Fig. 1e).

### Lead benzoxaboroles are prodrugs activated by a parasite serine carboxypeptidase

**Fig. 2:**
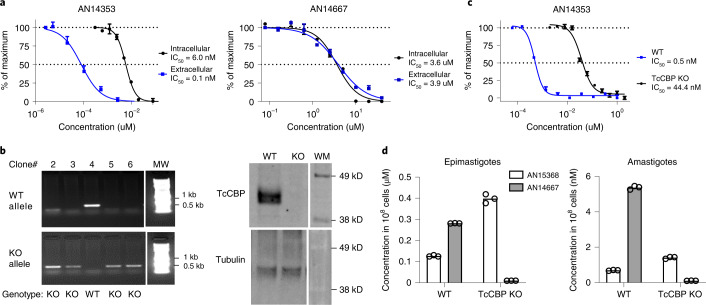
*T. cruzi* serine carboxypeptidases activate lead benzoxaborole compounds. **a**, IC_50_ of parent compound AN14353 and predicted peptidase cleavage product AN14667 on intracellular and extracellular amastigotes. Three replicates were performed at each concentration, assays were repeated >3 times. **b**, Left: generation of CBP KO lines in epimastigotes. WT and KO alleles were amplified using gene-specific primers (see Methods). Clones with only KO but not WT allele are considered to be KO lines. Right: western blot validated the absence of TcCBP protein in the KO line using a TcCBP-specific antibody and tubulin detection as a control; TcCBP protein and tubulin were detected in different gels. **c**, Impact of CBP disruption on activity of AN14353 on extracellular amastigotes. **d**, Uptake and conversion of AN15368 to CBP cleaved product AN14667 in WT and CBP-disrupted epimastigotes (left; treated with 10 uM AN15368) and amastigotes (right; treated with 50 nM AN15368) of *T. cruzi*. Data are presented as mean ± s.e.m.; for **b** and **c**: *n* = 3 biological replicates; for **d**: data are presented as individual values of 3 technical replicates. Source data

We next sought to understand the essentiality of the ester group for activity, as this functional group carries liabilities for hydrolytic and metabolic instability. An early indication of the importance of the ester function to anti-*T. cruzi* activity was evident from the already noted lack of potency of the *t*-butyl ester AN15134. Amide, *N*-methyl amide, ketone, ether and acylsulfonamide analogues of AN11735, as well the 1,2,4-oxadiazole ester bioisostere AN14562 all lacked activity (Supplementary Table 4). Furthermore, the expected carboxylic acid metabolite, AN14667, had ~1,000-fold reduced activity on both intra- and extracellular amastigotes (Figs. 1a and 2a). Thus, the ester functionality is absolutely essential for anti-*T. cruzi* activity.

We hypothesized that the esters might be prodrugs to the carboxylic acid and were cleaved within either the host cell or by a parasite enzyme. The high sensitivity of extracellular amastigotes to AN14353 but not to the carboxylic acid AN14667 (Fig. 2a) virtually eliminated the requirement for a host peptidase in prodrug activation. Several candidate enzymes with potential ester cleavage activity in *T. cruzi* were identified, including a serine carboxypeptidase (CBP; TcCLB.508671.20) present at 2–18 copies in different *T. cruzi* strains^14^ and a metallocarboxypeptidase 2 (TcCLB.504045.60). CRISPR/Cas9-driven disruption of all copies of the serine carboxypeptidases (Fig. 2b,c) but not the metallocarboxypeptidase (Supplementary Fig. 1) decreased in vitro sensitivity of *T. cruzi* amastigotes by up to 100-fold. Accumulation of the AN14353 analogue, AN15368, and its conversion to the cleaved product in wild-type *T. cruzi* epimastigotes and amastigotes but not in the TcCLB knockout (KO) line was documented by quantitative liquid chromatography tandem mass spectrometry (Fig. 2d), thus confirming the serine carboxypeptidase-dependent prodrug to drug conversion within *T. cruzi*.

### Mouse test of cure studies

**Fig. 3:**
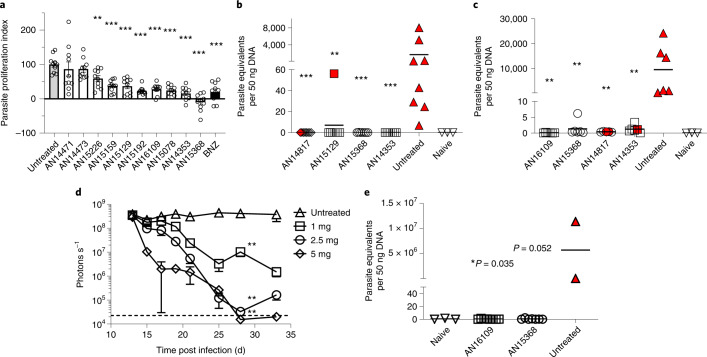
AN15368 cures *T. cruzi* infection in mice. **a**, Fluorescence intensity of C57BL/6J mice infected in the footpads with 2.5 × 10^5^ tdTomato-expressing *T. cruzi* was measured by in vivo imaging directly before (2 dpi) and 2 d after (4 dpi) a single oral dose of the compounds (50 mg kg^−1^). The parasite proliferation index after treatment was calculated as described in Methods (*n* = 10 individual feet). **b**, A daily oral treatment of the indicated compounds (10 mg kg^−1^) was administered for 40 d to C57BL/6J mice acutely infected with *T. cruzi* beginning at 17 dpi. Mice were immunosuppressed at the end of the treatment and samples were collected for parasitemia, haemoculture and qPCR of skeletal muscle (untreated, AN14817 and AN15129: *n* = 8; AN15368 and AN14353: *n* = 7; naive: *n* = 3 mice). **c**, Acutely infected mice (20 dpi) were treated orally with 10 mg kg^−1^ of compounds for 20 d. Parasite load in skeletal muscle was quantified by qPCR following immunosuppression at the end of the treatment. Dotted line represents the cut-off for the qPCR assay (untreated, AN16109, AN15368 and AN14353: *n* = 6; AN14817: *n* = 5; naive: *n* = 3 mice). **d**, On days 13–33 post infection, oral doses of AN15368 were administered at the indicated concentrations to hairless mice infected i.p. with 5 × 10^4^ luciferase-expressing *T. cruzi*. Bioluminiscence signals from whole mouse ventral images were acquired and quantified throughout the treatment by in vivo imaging (untreated, 7 mice; treated, 5 mice). Dashed line indicates background level in uninfected mice. **e**, C57BL/6J mice infected with 10^4^ *T. cruzi* i.p. were treated daily for 40 d, starting at 15 dpi with AN16109 or AN15368 (both at 2.5 mg kg^−1^). Parasite load was determined by qPCR of skeletal muscle samples after immunosuppression and blood samples were analysed by light microscopy and haemoculture (AN16109: *n* = 8; AN15368: *n* = 7; naive: *n* = 3; untreated: *n* = 2 mice). Red symbols denote animals in which parasites were detected by microscopy and/or haemoculture. Data are presented as mean ± s.e.m. Asterisks represent the statistical significance of the difference between each treated group and the untreated control. For **a**, **b** and **c:** Mann-Whitney test, two tailed; for **e**: unpaired *t*-test, 95% confidence interval. *P ≤ 0.05; **P ≤ 0.01; ***P ≤ 0.001. Source data

Evaluation of dose proportionality of exposure with AN14353 and generation of the carboxylic acid metabolite AN14667 in vivo (Supplementary Fig. 2) suggested solubility-limited absorption of this compound, prompting us to attempt to further optimize aqueous solubility. Focusing on the ester region, a variety of more polar, non-basic substituents such as aliphatic and cyclic ethers as well as hydroxyvaline analogues (predicted to be less hydrophilic) of our lead compounds were evaluated for both solubility and in in vitro trypanocidal assay (Supplementary Table 5). The highest in vitro-active compounds in this set were then evaluated and found to have anti-*T. cruzi* activity in the 2 d in vivo assay (Fig. 3a). Extensive in vitro and in vivo pharmacokinetic analysis and metabolic stability assays in mouse and human liver S9 fraction, and both mouse and human plasma and in vivo pharmacokinetics studies (summarized in Supplementary Table 6) ultimately identified three additional compounds of particular interest—AN14817, AN15368 and AN16109.

On the basis of these aggregate data, we progressed these and related compounds into a series of ‘test of cure’ assays in mice with a terminal immunosuppression period to reveal residual infection^5^. An initial screening using 10 mg kg^−1^ for 40 d identified several compounds for which the treated animals showed no parasite recovery by haemoculture and no parasite DNA detection in skeletal muscle using PCR (Fig. 3b). A more stringent test using only 20 d of treatment (a treatment period for which benznidazole is only partially effective in generating cure^5^) further distinguished the highest potency compounds from less potent ones (Fig. 3c). On the basis of these results, lower doses of the candidates were evaluated in a short-term (non-cure) treatment course of intraperitoneal infection with luciferase-expressing parasites (Fig. 3d and Supplementary Fig. 3) from which a dose of 2.5 mg kg^−1^ was identified and shown effective in a 40 d treatment cure assay (Fig. 3e).

### Non-human primate test of cure study

AN14353, AN14817 and AN15368 were progressed to an array of preliminary safety pharmacology, genotoxicity and toxicology studies. All three compounds were found to exhibit little to no affinity for a broad array of mammalian enzymes, receptors and ion channels, were non-genotoxic in standard Ames test and in vitro micronucleus studies, and did not demonstrate marked inhibition of representative cytochrome P450 enzymes at 10 uM. High-dose 7 d toxicology studies did not distinguish the three compounds from each other, but the non-dose proportional exposure noted previously with AN14353 (Supplementary Fig. 2) was also seen with the benzylic ester AN14817, probably a consequence of solubility-limited oral absorption. In contrast, the more hydrophilic analogue AN15368 exhibited good dose proportional exposure in rats (Supplementary Fig. 4) and modest effects on haematology and clinical chemistry at 150 mg kg^−1^, but none at 120 mg kg^−1^ d^−1^ or lower. Total plasma exposure (AUC_0–24 h_) in rats at 120 mg kg^−1^ d^−1^ was approximately 30,000 ng h ml^−1^, with no evidence of drug accumulation between the first and seventh days of the study. On the basis of these observations, AN15368 was selected as a pre-clinical candidate for the treatment of Chagas disease and for evaluation in rhesus macaques (*Macca mulatta*) infected with *T. cruzi* via natural exposure in the United States.

**Fig. 4:**
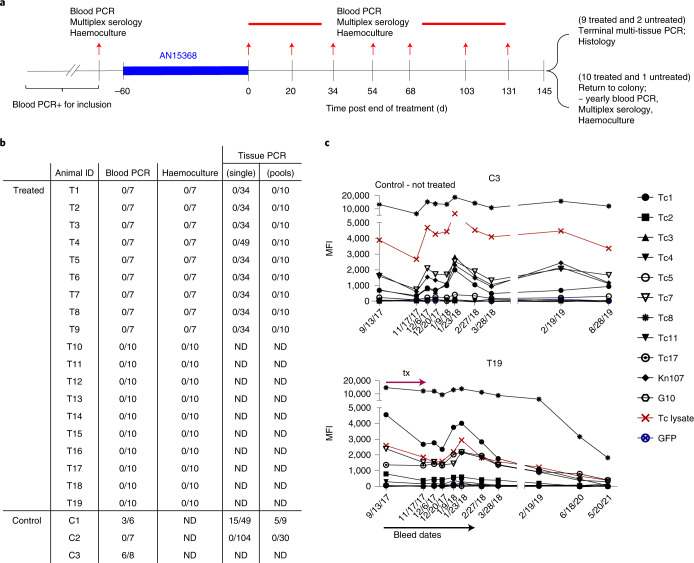
AN15368 uniformly cures chronically infected rhesus macaques. **a**, Structure of drug trial with indicated treatment period and sampling dates. **b**, Summary of post-treatment detection of infection using PCR of parasite DNA in blood or tissue (either from individual tissue samples or pools of 5 individual samples/determination; see Methods and Supplementary Tables 8–10 for details) and haemoculture in drug-treated (T1–T19) and untreated control (C1–3) macaques. Samples were taken at the 7 time points indicated in **a** and yearly thereafter for animals T1–T19 that were not euthanized and thus not tissue sampled for PCR. Fractions in the table (for example ‘0/7’) indicate the number of positive results per total number of samples analysed. **c**, Representative pre- and post-treatment IgG responses to recombinant *T. cruzi* proteins in control animal C3 and treated animal T19 (see Methods for identification and Extended Data Fig. 3 for remaining animals). MFI, mean fluorescence intensity. ND, not done. Source data

We chose to treat NHP for 60 d as this is the standard length of treatment employed for human infections and in previous clinical trials^2,3^ (Fig. 4a). On the basis of pharmacokinetic studies in NHPs (Supplementary Fig. 5) and allometric scaling^15^ (see Methods for additional details), a dose of 30 mg kg^−1^ was chosen for NHP to provide a high possible rate of cure without compromising safety. To maximize the power of detecting an expected high rate of cure, 19 animals were enrolled in the single-arm treatment study and 3 animals served as untreated controls. Pre-treatment data on the animals are shown in Supplementary Table 8; the mean age of the animals in the treatment group was 19.4 yr and the average minimal length of infection was 5.7 yr.

**Extended Data Fig. 2:**
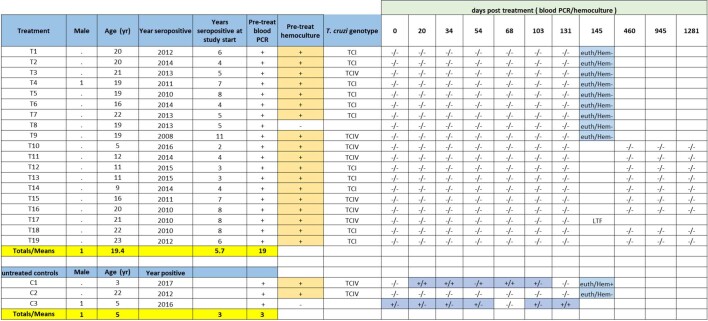
Pre-, and post-treatment infection status of NHP. Euth/Hem = hemoculture results of blood collected at time of euthanasia. LTF = lost to follow-up due to the animal’s death from traumatic lesions it acquired through conspecific interactions.

**Extended Data Fig. 3:**
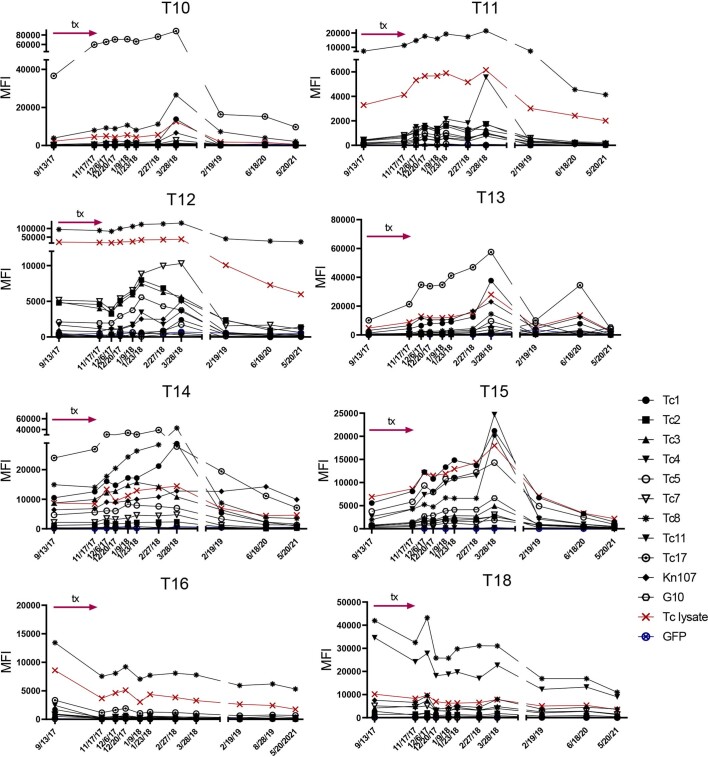
Declining IgG levels to recombinant *T. cruzi* proteins over time in AN15368-treated NHP. Luminex-based pre- and post-treatment IgG responses to recombinant *T. cruzi* proteins in AN15368-treated macaques not pictured in Fig. 4 (see Methods for identification of recombinant proteins). Source data

All animals in the trial were positive by PCR for *T. cruzi* DNA in blood at one or more time points pre-treatment and were seropositive for anti-*T. cruzi* antibodies by standard facility screening tests and via our Luminex-based multiplex serological assay^16,17^ (Extended Data Figs. 2 and 3, and Fig. 4). Parasites were also isolated from haemocultures of pre-treatment blood samples from 18 of the 19 animals in the treatment group and 2 of 3 control animals. Importantly, the isolated parasite lines showed a variability in genetic types (DTU), indicating a diversity of parasite lineages in these animals, even though they acquired infection in the relatively restricted geographic footprint of the primate colony (Extended Data Fig. 2). These latter characteristics are similar to those found in a previous US-based study in cynomolgus macaques with naturally acquired *T. cruzi* infection^17^.

The primary endpoints of the trial were detection of parasite DNA in blood and culture of parasites from blood, for which all animals in the study were assayed a minimum of 7 times at 2–4.5-week intervals following the end of treatment (Fig. 4a). All AN15368-treated animals were negative by both assays at all time points (Fig. 4b and Supplementary Table 7). In contrast, 2 of the 3 untreated animals were positive by one or both haemoculture and PCR at multiple time points.

A secondary determinant of treatment efficacy was the detection of *T. cruzi* DNA by PCR in post-necropsy tissues. For this purpose, 9 of the 19 treated animals and 2 of the untreated controls were euthanized and tissues collected. DNA was extracted and analysed by PCR for *T. cruzi* kinetoplast DNA (kDNA) using both individual and pooled tissue samples (Fig. 4a,b and Supplementary Table 8). For control animal C1, 30 of 68 (39.4%) single or pooled tissue samples from multiple tissues yielded positive PCR results. In contrast, for the 9 treated animals, tissue samples assayed singly or in pools (range 84–99 total sample sites per animal) from heart, quadriceps, biceps, small and large intestine, oesophagus, tongue, liver, spleen, abdominal fat and brain were all negative. Interestingly, we also failed to detect parasite DNA in non-treated control animal C2 by blood or tissue PCR despite its being positive by blood PCR and haemoculture in pre-treatment sampling.

A third measure of treatment efficacy was declining antibody levels to a set of recombinant *T. cruzi* proteins in the multiplex serological assay^16,17^. Monitoring for decreases in anti-*T. cruzi* antibodies has been useful for assessing treatment efficacy in humans and conversion to seronegative is considered the standard for determining infection cure, although in many cases this can take years post-treatment to achieve^18–20^. Nine of the remaining 10 treated macaques not killed at the end of treatment were returned to the breeding colony and thus were available for continued periodic monitoring over >3 yr (Fig. 4a). All animals showed declines in antibody levels to multiple recombinant proteins from *T. cruzi* over the 42 months after the end of treatment (Fig. 4c and Extended Data Fig. 3). Importantly, blood PCR and haemoculture samples at these additional post-treatment time points also remained negative in all treated macaques (Extended Data Fig. 2 and Supplementary Table 7). Thus, the exhaustive examination of blood and tissues for parasite DNA and long-term monitoring of anti-*T. cruzi* antibodies conclusively establish a 100% efficacy of AN15368 in a population of time-variable, naturally infected NHPs harbouring genetically diverse populations of *T. cruzi*.

Throughout the dosing period, macaques readily accepted food treats containing compound and no post-dose nausea or other interruption of normal activity was observed. No adverse events were noted in any of the 19 treated macaques during the 60 d treatment period and repeated physical examinations revealed no clinical signs that could be associated with drug administration. According to the blood-based health screening performed throughout the study, the mean values of the liver enzymes alanine aminotransferase and alkaline phosphatase, and the levels of lymphocytes and monocytes in the blood were mildly elevated during the drug-treatment phase of the study and returned to pre-study values by the final blood draw at the end of the study (Supplementary Data 1). The 9 female animals that returned to the breeding colony have shown no abnormalities in the yearly examinations and have produced 13 healthy and *T. cruzi*-seronegative infants in the first 2 yr following treatment. These latter numbers are wholly consistent with the fecundity rate of the colony in general. At necropsy, 3 of 11 euthanized animals (2 treated animals and 1 control) were identified on gross examination to have pale areas in the heart that could be consistent with myocardial damage associated with Chagas disease. Histological examination revealed inflammation in several cases but did not differentiate the treated from untreated (control) study animals and no *T. cruzi* amastigotes were detected in any tissues from the study animals (Supplementary Data 1). Thus, AN15368 is both highly effective in curing long-standing *T. cruzi* infections and presents no overt safety or reproductive health concerns in a 60 d treatment course in NHPs.

### Target identification

**Fig. 5:**
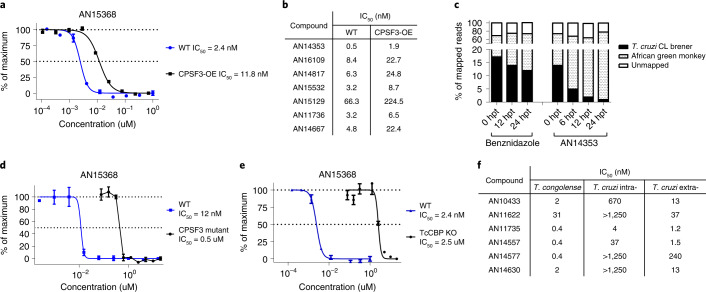
AN15368 is activated by a *T. cruzi* serine carboxypeptidase and targets CPSF3. **a**,**b**, Overexpression of CPSF3 increases resistance to AN15368 (**a**) (CPSF3-OE: *n* = 3 biological replicates; WT: *n* = 2 biological replicates), as well as to related benzoxaboroles (**b**). All assays were carried out in intracellular amastigotes. **c**, Suppression of parasite mRNA relative to host cell (African green monkey (Vero)) mRNA by exposure to AN14353 as indicated by the decline in the fraction of sequence reads mappable to parasite genome relative to host cell genome (hpt, hours post-treatment). **d**, Engineered Asn to His mutation at amino acid position 232 of CPSF3 conveys resistance to AN15368 by intracellular amastigotes (*n* = 3 biological replicates). **e**, Disruption of CBPs leads to a 1,000× increased resistance to AN15368 in intracellular amastigotes (TcCBP KO: *n* = 3 biological replicates; WT: *n* = 2 biological replicates). **f**, Some AN15368 analogues lacking activity to intracellular *T. cruzi* (intra-) are effective killers of the *T. congolense* (extracellular) and *T. cruzi* extracellular amastigotes (extra-). Data are presented as mean ± s.e.m. Source data

**Extended Data Fig. 4:**
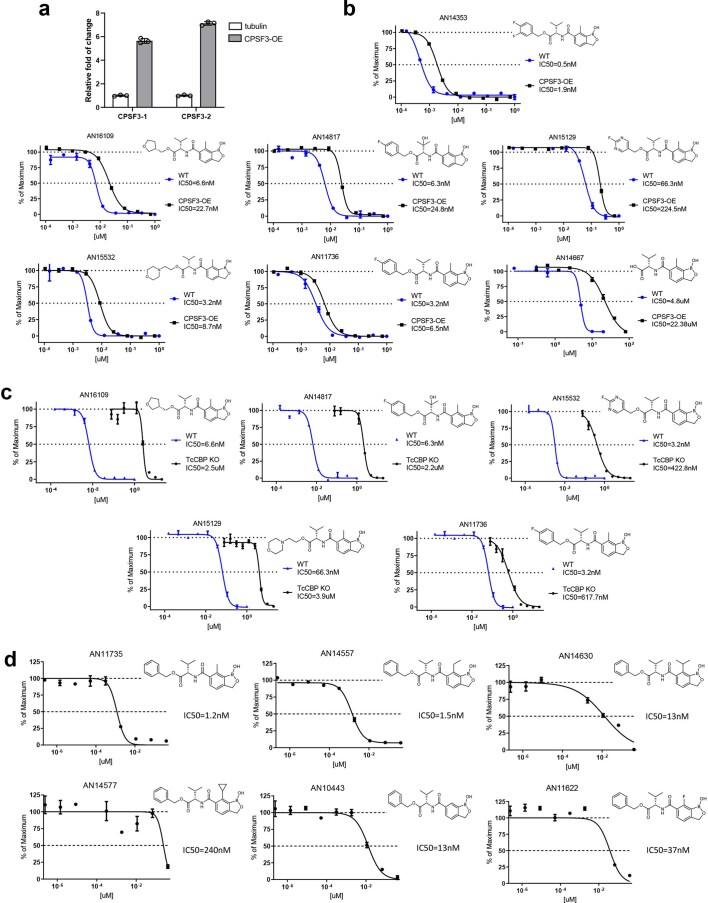
AN15368 is activated by a *T. cruzi* serine carboxypeptidase and targets CPSF3. **a**. Fold change in CPSF3 transcripts by qRT-PCR in two CPSF3-overexpressing *T. cruzi* lines (n = 3 biological replicates). **b**. Overexpression of CPSF3 in *T. cruzi* results in increased resistance to all AN15368 analogues (CSPF3-OE: n = 3 biological replicates; WT: n = 2 biological replicates). **c**. AN15368 analogues require activation by the *T. cruzi* CBP in order to efficiently kill intracellular *T. cruzi* as demonstrated by the increased resistance of CBP deficient parasites (TcCBP KO: n = 3 biological replicates; WT: n = 2 biological replicates). **d**. AN15368 analogues with poor activity against intracellular *T. cruzi* amastigotes have low nanomolar activity on extracellular amastigotes (n = 3 biological replicates). Data are presented as mean values + /- SD. Source data

During the course of this work, several benzoxaborole analogues of AN15368 with efficacy in the treatment of African trypanosomiasis in cattle were shown to target the Cleavage and Polyadenylation Specificity Factor (CPSF3), an important factor in messenger RNA processing^21,22^. Similar to findings reported in these studies, the overexpression of CPSF3 in *T. cruzi* (Extended Data Fig. 4) also resulted in a 3–5-fold increase in resistance to AN15368 (Fig. 5) as well as cross resistance to other benzoxaborole analogues (Fig. 5b and Extended Data Fig. 4), suggesting that CPSF3 is at least one of the targets of AN15368 in *T. cruzi*. This conclusion is also supported by the marked and continuing reduction in parasite mRNA as early as 6 h following addition of AN14353 to *T. cruzi*-infected host cells, but not in benznidazole-treated cultures (Fig. 5c). Furthermore, introduction into *T. cruzi* of the Asn232 mutation to His in CPSF3, reported by ref. ^21^ to disrupt binding of benzoxaborole compounds in *T. brucei brucei*, also conferred resistance to AN15368 (Fig. 5d). Lastly, and as noted above for AN14353 (Fig. 2), AN15368 and other related analogues all function as prodrugs and require activation by the *T. cruzi* CBP to efficiently kill intracellular *T. cruzi* (Fig. 5e and Extended Data Fig. 4c), as is the case for the benzoxaboroles effective against African trypanosomes^23^. Thus, the highly effective benzoxaborole AN15368 is a prodrug that enters host cells and then *T. cruzi*, wherein it is cleaved by a *T. cruzi* peptidase. The product of this cleavage selectively targets CPSF3-mediated mRNA maturation in intracellular amastigotes.

Although the target of these benzoxaboroles in *T. cruzi* and in African trypanosomes appears to be the same, a number of the compounds with previously reported potent activity in vitro to *T. congolense* had activity on extracellular amastigotes but not on *T. cruzi* amastigotes in host cells (Fig. 5e and Extended Data Fig. 4d). Reasoning that this differential effect could be due to variable or limited entry into or metabolism by *T. cruzi* host cells, we screened for the activity of these compounds on extracellular amastigotes of *T. cruzi*. With one possible exception, all of these compounds have low nanomolar activity on extracellular amastigotes, indicating an additional selectivity of benzoxaborole activity on *T. cruzi* due to this parasite’s intracellular lifestyle.

## Discussion

The benzoxaborale AN15368 is the first highly effective compound for the treatment of *T. cruzi* infection discovered in >50 yr, and the only compound so far shown to achieve unequivocal and apparently uniform cure of infection in NHP with long-term naturally acquired infections of diverse *T. cruzi* genetic types. AN15368 is orally bioactive and exhibited no overt toxicity in a 60 d course of treatment in NHP. Thus, AN15368 is a very strong candidate for ultimately progressing into human clinical trials.

Despite some successful vector control efforts, the risk of infection with *T. cruzi* remains variable but substantial for human and other animals from the southern United States to southern South America. The currently available drugs benznidazole and nifurtimox suffer from variable efficacy and high rates of adverse events. Consequently, these drugs are not routinely used despite their relatively wide availability. The absence of highly effective treatments undermines the use of widespread and routine screening that would detect the usually asymptomatic early infection before irreversible damage is done^24^. A relatively large number of potential candidates have been targeted for development, some for decades (reviewed in ref. ^25^), but those that have been progressed to human clinical trials^2,3,26^ have performed significantly worse than currently available drugs.

The benzoxaboroles have become a rich source of development candidates for treatment of protozoal infections, with an apparent target of all being the mRNA-processing endonuclease, CPSF3^10,21–23,27–31^. Although it was initially hypothesized that AN15368 and the analogue AN11736 may not target CPSF3 on the basis of their limited effect on mRNA processing^22^, subsequent work demonstrated that overexpression of CPSF3 in *T. b. brucei* induced resistance to killing by AN11736^21^ and we arrive at a similar conclusion with respect to the activity of AN15368 in *T. cruzi*, indicating CPSF3 as one probable target. Also, similar to a number of benzoxaboroles highly effective against the African trypanosomes, AN15368 requires processing into its carboxylate form to achieve full potency and we show that this activation is mediated by a parasite serine carboxypeptidase. However, unlike the case in the extracellular African trypanosomes, AN15368 must traffic unprocessed through both the host and the parasite plasma membranes to reach these activating enzymes. This requirement appears to account for the differential activity of a number of highly similar benzoxaboroles on African trypanosomes and *T. cruzi*, and emphasizes the need to tailor drugs to match the specific biology of the pathogen, even when the processing/activation requirements and the target of the compounds are the same. Likewise, this outcome highlights the challenge of designing compounds with cross-species activity for genetically related but biologically diverse pathogens such as the kinetoplastids.

The differential dosing requirements for the benzoxaboroles in *T. cruzi* infection, where 20 or more days of treatment is necessary for sterile cure as compared with African trypanosomes where a single dose effects cure in cattle^10^, is remarkable and further underscores the difficulties of drug discovery for *T. cruzi*. *T. cruzi* can invade diverse host cell types in tissues throughout the body, presenting a challenge for any one drug to reach effective levels in all tissues. Furthermore, *T. cruzi* amastigotes have recently been shown to assume a non-dividing, apparently low-metabolic state that provides substantial resistance to drugs^4,32^. Fortunately, these properties do not prevent drug-induced sterile cure but appear to necessitate an extended treatment course, as observed herein, and previously for other anti-*T. cruzi* drugs^32^. The NHP trial conducted as part of this study was initiated before knowledge of this dormancy property in *T. cruzi* and thus utilized daily dosing for 60 d, as is common for the currently used benznidazole and nifurtimox. Despite these lengthy treatment periods, drug-induced resistance in *T. cruzi* has neither been reported with respect to benznidazole and nifurtimox nor have we observed resistance during the extended treatment courses using benzoxaboroles, even though all three drugs are produgs. Nevertheless, shortened or modified treatment regimens may be possible with the benzoxaboroles (Fig. 3c) as is the case for benznidazole^5,32,33^, to further reduce this possibility. As a prodrug, AN15368 has the liability of potential selection of resistance via loss of the processing carboxypeptidase^23^, although deletion of the CBP array in *T. cruzi* substantially reduces but does not totally abolish susceptibility to AN15368.

One noteworthy advantage of drug discovery in *T. cruzi* is the very wide natural host range of the parasite, including most wild and domesticated mammals as well as mice, canines and NHP, in addition to humans. In all these hosts, *T. cruzi* appears to behave similarly, infecting the same host cell types, being controlled (but rarely eliminated) by similar immune effector mechanisms, generating analogous pathologies, and being affected correspondingly by the same drugs. Animals in *T.* cruzi-endemic areas, including the southern United States, are at risk of acquiring *T. cruzi* infection and in some areas, this risk is severe, leading to 20–30% new infections per year in some populations^34^ as well as infections in zoo animals^35,36^. The commonly used indoor/outdoor housing of NHPs in *T. cruzi*-endemic regions also results in naturally acquired *T. cruzi* infection in animals in these facilities, despite vector control and other preventative measures^37^. Infections in these NHPs mirror that in humans, initiating at different points in life and extending for decades in some cases, involving genetically and phenotypically diverse parasite populations, and leading to a diversity of immune responses and disease outcomes^17^. All these characteristics make these NHPs incredibly valuable resources for trialling anti-*T. cruzi* drugs before human clinical trials. The observed 100% cure with AN15368 in macaques harbouring long-term infections with genetically diverse parasite lineages and without any apparent drug toxicity bodes well for the potential safety and efficacy of AN15368 in humans. It is noteworthy that the only other documented treatment trials in *T. cruzi* -infected NHPs recorded a high degree of failure for posaconazole (100%)^38^ and benznidazole (>60%; R.L.T. et al., manuscript in preparation), in agreement with the high failure rate of these drugs in human clinical trials^2,3,26^. We also validate the same methods used for monitoring treatment efficacy in humans, specifically serial blood PCR for *T. cruzi* DNA and changes in immune profiles, for use in NHPs and reinforce these metrics with extensive tissue PCR in a subset of necropsied animals. Lastly, the ability to return infection-cured macaques to the breeding colony extends the utility of these resources, providing the potential to monitor disease development and the susceptibility to reinfection, for example, in hosts previously cured of *T. cruzi* infections.

Surprisingly, we also observed one apparent self-cure among the three untreated NHPs. Although documentation of spontaneous cures is relatively rare^39–41^, they are not without precedent and may occur more frequently than currently appreciated^42^ but have not been previously observed in this NHP colony.

The success of this project provides insights into some best practices for drug discovery in *T. cruzi* and perhaps related organisms. In addition to taking advantage of the potency of the oxaboroles in general as anti-infectives, and the med-chem knowledgebase in this class of compounds, this project also benefitted substantially from the accessibility of infection systems for screening and testing compounds. Specifically, the power of the mouse-based assays to quickly, easily and quantitatively assess in vitro-active compounds for in vivo activity was instrumental in rapidly identifying the compounds with the highest potential. Coupled with the availability of substantial numbers of NHPs with naturally acquired *T. cruzi* infections for pre-clinical validation, we should be able to avoid the clinical trial failures that have accompanied previous drug discovery efforts in Chagas disease.

## Methods

### Compound synthesis

All compounds used in this study were prepared as described in US patent 10,882,272 granted on 5 January 2021. The syntheses of representative compounds AN14353 and AN15368 are described in Supplementary Text 1.

### Parasites and mice

C57BL/6J mice were purchased from the Jackson Laboratory and B6.129S7-Ifngtm1Ts/J (IFN-gamma deficient) were bred in-house at the University of Georgia Animal Facility. The SKH-1 ‘hairless’ mice backcrossed to C57BL/6J were a gift from Dr Lisa DeLouise (University of Rochester). All the animals were maintained at the University of Georgia Animal Facility under specific-pathogen-free conditions at 22 °C, 50% humidity and in a 12:12 h light:dark cycle. Male and female mice 6–9 weeks of age were used. All mouse experiments were carried out in strict accordance with the Public Health Service Policy on Humane Care and Use of Laboratory Animals and the Association for Assessment and Accreditation of Laboratory Animal Care accreditation guidelines. The protocol was approved by the University of Georgia Institutional Animal Care and Use Committee (IACUC). *T. cruzi* tissue culture trypomastigotes of the wild-type Brazil strain, Colombiana strain co-expressing firefly luciferase and tdTomato reporter proteins^4^, and CL strain expressing the fluorescent protein tdTomato^11^ were maintained through passage in Vero cells (American Type Culture Collection) cultured in RPMI 1640 medium with 10% fetal bovine serum at 37 °C in an atmosphere of 5% CO_2_. Parasite genotypes were determined as previously described^17^.

### In vitro amastigote growth inhibition and killing assays

The in vitro anti-*T. cruzi* amastiogote activity assay was performed and optimized on the basis of the protocol described previously^11^. The change in tdTomato fluorescence intensity was determined as a measurement of growth over 72 h of culture. For assaying drug effects on extracellular amastigotes, trypomastigotes were collected from infected Vero cell cultures and converted in acidic media as previously described^43^. Amastigotes (50,000 per well) were incubated with 2-fold serial dilutions of compounds for 48 h. ATP production was used as an indication of growth in this case and was measured by ATPlite Luminescence ATP detection assay system (PerkinElmer). Both fluorescence and luminescence were read using a BioTek Synergy hybrid multi-mode reader equipped with the software Gene5 v 2.0 (BioTek). The dose-response curve was generated by linear regression analysis with GraphPad Prism v9.4.0 (GraphPad software). IC_50_ was determined as the drug concentration that was required to inhibit 50% of growth compared to that of parasites with no drug exposure.

### In vivo compound screens

#### Rapid assays

C57BL/6J mice were injected in the hind footpads with 2.5 × 10^5^ tdTomato-expressing *T. cruzi* (CL strain) and orally treated with a single dose of the compounds (50 mg kg^−1^) at 2 days post infection (dpi). Fluorescent intensity of the feet was measured at 2 dpi before compound administration and at 4 dpi in the Maestro in vivo imaging system equipped with the software Maestro 2.1.0 (CRi) as previously described^44^. The proliferation index was estimated as PI = [(T4d – T2d)/(mUnt4d − mUnt2d)] × 100; where T4d and T2d are the fluorescence intensity of the feet of the treated animals at days 4 and 2 post infection, respectively; mUnt4d and mUnt2d are the average fluorescence intensity of the feet of the untreated animals at 4 and 2 dpi, respectively.

#### Cure assays

Male or female C57BL/6J mice were intraperitoneally infected with 10^4^ trypomastigotes of the Brazil strain. Mouse infection was confirmed at 25–30 dpi by detection of CD8^+^ T cells specific against the *T. cruzi* TSKb20 peptide in blood^45^. Compounds resuspended in 1% carboxymethyl cellulose and 0.1% Tween 80 were administered daily by gavage at the specified concentrations. To optimally detect persistent infection, immune responses in the mice were suppressed by intraperitoneal (i.p.) injection of four doses of cyclophosphamide every 2–3 d (200 mg kg^−1^ d^−1^), beginning at 1 week after the end of therapeutic treatment. At the end of the immunosuppression regimen, peripheral blood was checked under light microscope for parasites and cultured in LDNT media^5^. Mouse skeletal muscle samples were obtained at the end of the immunosuppression and processed for *T.* cruzi DNA detection by qPCR as previously described^32^.

#### In vivo killing time assays

C57BL/6J mice were infected with 2.5 × 10^5^ luciferase-expressing *T. cruzi* (Brazil strain) in the footpads, and 2 d later one oral dose of AN14353 (25 mg kg^−1^) was administered. The bioluminescent signal in the feet after i.p. injection of d-luciferin (PerkinElmer; 250 mg kg^−1^) was measured in a Lumina II IVIS imager (PerkinElmer).

#### Low-dose short treatment

Hairless mice (SKH-1) were infected intraperitoneally with 5 × 10^4^ luciferase-expressing *T. cruzi* (Colombiana strain) and orally treated from 12 to 22 dpi with 1, 2.5 or 5 mg kg^−1^ of AN16109 or AN15368. The bioluminiscence signal of the whole body was measured after d-luciferin injection in a Lumina II IVIS in vivo imager equipped with the Living Imaging 4.0 software (PerkinElmer).

### Generation of CBP knockout

The CBP knockout was produced using ribonucleoprotein (RNP) complexes as previously described^46^. Briefly, Brazil tdTomato strain epimastigotes were electroporated with RNP complexes containing SaCas9 and a single-guide RNA (sgRNA) targeting the CBP gene TcBrA4_0048170, plus a repair template containing stop codons in all three reading frames and the M13 sequence for use as a PCR anchor. The sgRNA targeted the ‘GATTTACGTTGACCAGCCTGC’ sequence. After 2 d of recovery, single-cell clones were derived by depositing epimastigotes into a 96-well plate at a density of 0.5 cells per well by using a MoFlo Astrios EQ cell sorter (Beckman Coulter). DNA isolation was performed for clones, and the primer pair 5’-ACGTTGACCAGCCTGCAG-3’ and 5’- TGTGTATGGGTCTGTGAG-3’ was used for amplifying the wild-type allele, while the primer pair M13-F: 5’- TGTAAAACGACGGCCAGT-3’ and 5’-TGTGTATGGGTCTGTGAG-3’ was used for amplifying the mutant allele.

### Western blot

A total of 5 × 10^7^ epimastigotes were collected at 4 °C and washed once with cold PBS. Pellets were suspended in RIPA buffer (150 mM NaCl, 20 mM Tris HCl pH7.5, 1 mM EDTA, 1% SDS, 0.1% Triton X-100) with 1% protease inhibitor cocktail (Thermo Fisher) and incubated for 1 h on ice. Then the suspension was sonicated (Sonics & Materials, model 501) for 10 s using microtip probe at 25 amplitude, and the sonicate centrifuged at 16,000 *g* for 10 min to remove the pellets and obtain total protein. Western blot was performed according to the general established protocol. TcCBP-specific antibody (a gift from Drs Juan José Cazzulo and Gabriela Niemirowicz at Instituto de Investigaciones Biotecnológicas, Buenos Aires, Argentina) was diluted at 1:500 and β-tubulin antibody was diluted at 1:1,000. The IRDye 800CW donkey anti-rabbit IgG (Li-COR) was used as secondary antibody for both TcCBP and tubulin at 1:10,000 dilution. Images were taken with the Bio-Rad ChemiDoc imaging system with the software ImageLab Touch 2.4.03.

### Generation and confirmation of the CPSF3 overexpression line

The *CPSF3* gene was amplified using primers ‘ATGCTCCCTGCGGCAGCAGCAGTAA’ and ‘TTACACAGCCTCCTCTGGCAAAGGCT’, and integrated into the pTrex vector^47^ by NEBuilder HiFi DNA assembly (New England Biolabs). The construct pTrex-CPSF was then transfected into Brazil tdTomato epimastigotes and selected by 60 ug ml^−1^ blasticidin.

To confirm the CPSF overexpression in the selected transfectants, RNA was extracted as previously described^14^ and converted to complementary DNA (cDNA) using SuperScript reverse transcriptase (Invitrogen). Quantitative PCR reactions were performed in triplicate on the C1000 Touch Bio-Rad CFX96 real-time PCR detection system for CPSF using primer sets CPSF-1 (5’-TGAAACAGCAGCATGCCAAC-3’ and 5’-CGCGTCTGTCTACCATCAGA-3’) and CPSF-2 (5’-CGGCTCATTCTGATGGTAGACA-3’ and 5’-TGTGCGTTGCACACTGAATG-3’) in both control and CPSF overexpressing parasites. The expression level was normalized to tubulin and amplified using primers ‘AAGTGCGGCATCAACTACCA’ and ‘ACCCTCCTCCATACCCTCA’.

### Generation of CPSF3 mutants

To generate CPSF3 mutants, an RNP complex was transfected into Brazil tdTomato strain epimastigotes to target the *CPSF3* gene (TcBrA4_0124800), together with a repair template that contained the mutation of Asn^231^ to His^21^. The sgRNA targeted the ‘TCTGATTGCGGAAAGCACAA’ site. After 24 h of recovery, 20 uM AN15368 was added to the parasites to select CPSF3 mutants that were resistant to drug treatment. The ultimate resistant parasites were validated to have acquired the Asn^231^His mutation in CPSF3 via sequencing.

### RNA-seq sample preparation, sequencing and analysis

Vero cells (10^6^) were infected with 10^7^ CL strain trypomastigotes of *T. cruzi* for 2 d before treating with either 5 uM benznidazole or 30 nM AN14353. The drug concentration used for treatment was set at 5 times the IC_50_. Samples were collected at several time points for RNA extraction as previously described^48^. Ribosomal RNA (rRNA)-depleted RNA library construction and RNA sequencing using Illumina Nextseq 75PE was carried out by the Georgia Genomics and Bioinformatics Core (GGBC, University of Georgia, Georgia). Illumina reads with mean quality lower than 30 (Phred Score based) were removed from analysis, then mapped to the CL Brener genome (TritrypDB release-33) and the African green monkey genome^49^ using the HiSAT software package v0.1.6^50^ with default parameters. The mapping rate was quantified by HTseq v0.6.1^51^.

### LC–MS/MS analysis of intracellular AN15368 and AN14667

Wild-type and peptidase-knockout *T. cruzi* epimastigotes (5 × 10^8^) were treated with AN15368 (10 uM) or with DMSO vehicle control for 6 h. The cells were then pelleted and resuspended in 100 µl PBS. The cell suspension was mixed with 200 µl acetonitrile and centrifuged at 735 *g* for 10 min at room temperature. After extraction, the supernatant was further diluted with methanol:water (30:70; v/v) containing 0.4 nM internal standard AN14817 to a concentration within the calibration range. Each sample was diluted in triplicate to provide technical replicates and the diluted sample (10 µl) was injected for subsequent LC–MS/MS analysis.

LC–MS/MS analysis was performed on a Waters ACQUITY I-Class UPLC system coupled to a Xevo TQ-S triple quadrupole mass spectrometer. An ACQUITY UPLC BEH C18 column (130 Å, 1.7 µm, 2.1 mm × 50 mm) was used for chromatographic separation, and the column temperature was 40 °C. The mobile phase consisted of water (A) and methanol (B), both containing 0.1% (v/v) formic acid. The following gradient elution was performed at a flow rate of 0.4 ml min^−1^: 0–0.5 min, 30% B; 0.5–3 min, 30–95% B; 3–4 min, 95% B; 4–4.1 min, 95–30% B; and 4.1–5 min, 30% B. The MS ionization was carried out in the positive electrospray ionization mode with following conditions: capillary voltage = 1.50 kV, desolvation temperature = 500 °C, desolvation gas flow = 1,000 l h^−1^ and nebulizer gas pressure = 7.0 bar. The MS/MS transitions used for detection and quantification were 390.1→174.9 for AN15368, 292.0→174.9 for AN14667 and 416.1→109.0 for AN14817. Data were processed using TargetLynx v4.1 software (Waters).

### NHP resource and facilities

All NHPs utilized for these studies were acquired from the approximately 1,000-animal Rhesus Macaque (*Macaca mulatta*) Breeding and Research Resource housed at the AAALAC accredited Michale E. Keeling Center for Comparative Medicine and Research (KCCMR) of The University of Texas MD Anderson Cancer Center in Bastrop, Texas. This is a closed colony, which is specific-pathogen-free (SPF) for Macacine herpesvirus-1 (Herpes B), Simian retroviruses (SRV-1, SRV-2, SIV and STLV-1) and *Mycobacterium tuberculosis* complex. All animals are socially housed in shaded, temperature-regulated indoor-outdoor enclosures with numerous barrels, perches, swings, and various feeding puzzles and substrates to mimic natural foraging and feeding behaviours. Standard monkey chow, ad libitum water and novel food enrichment items are provided daily. Study animals that were seropositive for *T. cruzi* had acquired the infection naturally through exposure to the insect vector of the parasite while in their indoor-outdoor housing facilities. The NHP experiments were performed at the KCCMR and all protocols were approved by the MD Anderson Cancer Center’s IACUC and followed the NIH standards established by the Guide for the Care and Use of Laboratory Animals^52^.

### Pharmacokinetic (PK) analysis in NHP

Pre-treatment PK analysis of AN15368 distribution and clearance was performed to assist in determining the treatment dosing regimen. While under sedation/general anaesthesia, AN15368 was administered at various dosing levels either intravenously (IV) or via oral gavage (PO) to a *T. cruzi* -seronegative rhesus macaque, and 500 ul blood samples were collected before dosing and at 2, 5, 15, 30 and 60 min post-dose administration at which time the animal was recovered from general anaesthesia. Additional 500 ul blood samples were collected at 3, 6, 9 and 24 h post-dose administration under light anaesthesia/sedation. After the initial IV/PO PK assessment, a pre-regimen PK assessment of oral dosing was conducted, with administration of a single dose of AN15368 in 3 animals over 3 dosing periods, with AN15368 (30 or 50 mg kg^−1^ dose) administered in food treats. For this pre-regimen phase, blood samples were collected at pre-dose, then at 0.25, 0.5, 1, 3, 6, 9 and 24 h post dose.

Mid- and end-regimen PK assessments were also performed. A ‘peak and trough’ mid-regimen (day 30) PK assessment was performed on 3 treated animals: (1) blood was collected before drug dosing, (2) the animals were gavage-dosed with AN15368 in pumpkin slurry and (3) a second blood sample was collected at 3 h post dosing. The end-regimen PK analysis was performed on the 60th (and final) day of AN15368 dosing, with a non-serial sparse sampling design utilized for 18 of the 19 treated animals. For this study, 3 animals had blood collected before provision of the AN15368 in food treats. The other 15 animals were provided AN15368 in food treats and then blood was collected from 3 separate animals at 0.5, 1, 3, 6 and 9 h post dosing. Only 1 blood sample was collected at a single time point from each of the 18 animals. The composite plasma PK profile on day 60 was obtained using the mean concentrations (*n* = 3) at each sampling time point (pre-dose, 0.5, 1, 3, 6 and 9 h). All blood samples (500 ul) for PK analysis (pre-, mid- and end-regimen) were collected into EDTA microtainers and plasma collected for the determination of AN15368. The plasma samples were provided to Pharmout Labs (Fremont, California) for analysis using LC–MS/MS. The mean pre-dose concentration was also depicted as the 24 h post-dose concentration and used for the calculation of the post-treatment AUC_0–24_ value on day 60.

For calculation of PK parameters, the *C*_max_ (maximum concentrations) and *t*_max_ (time to maximum concentrations) were determined by visual inspection of the plasma concentration vs time curves from the pre-regimen and end-regimen periods. PK calculation was not performed for the mid-regimen PK samples since only two time points were collected. The AUC values for the pre- and end-regimen were calculated using the linear-trapezoidal rule with the following equation:

$${{{{{\mathrm{AUC}}}}}}_{({{{t}}}1 - {{{t}}}2)} = [({{{C}}}_{{{{t}}}2} + {{{C}}}_{{{{t}}}1}) \times \left( {{{{t}}}2-{{{t}}}1} \right)]/2$$

where *t*1 and *t*2 are consecutive sampling time points, AUC_(*t*1-*t*2)_ is the fractional AUC over time intervals *t*1 and *t*2, *C*_*t*2_ is the concentration at time *t*2 and *C*_*t*1_ is the concentration at time *t*1. The total AUC (AUC_0–24_) over the dosing interval (24 h) was calculated by summation of all fractional AUC values over the intervals between 0 (pre-dose) and 24 h (post-dose).

When a terminal elimination phase was apparent in the plasma concentration vs time curve, the terminal half-life (*t*_1/2_) was estimated using the equation *t*_1/2_ = 0.693/*ƛ*_*z*_, where *ƛ*_*z*_ is the elimination rate constant estimated from the slope of the terminal elimination phase. AUC0- ∞ was estimated using the following equation:

$${{{{{\mathrm{AUC}}}}}}_{0 - \infty } = {{{{{\mathrm{AUC}}}}}}_{{{{{{\mathrm{last}}}}}}} + {{{C}}}_{{{{{{\mathrm{last}}}}}}}/{\bar{\lambda}}_{{{{{\mathrm{z}}}}}}$$

where AUC_0–∞_ is AUC from zero to infinity, AUC_last_ is the AUC from zero to the last measurable time point and *C*_last_ is the concentration at the last measurable time point. AUC_0–∞_ and *t*_1/2_ were not estimated when the terminal elimination phase was not defined.

Plasma clearance (CL_p_) after the IV dose was estimated using the following: CL_p_ = Dose/AUC_0–∞_. Bioavailability (%*F*) after a single oral dose was estimated using the following:

$$\% {{{F}}} = \left( {{{{{{\mathrm{AUC}}}}}}_{0 - \infty ,\,{{{{{\mathrm{PO}}}}}}}/{{{{{\mathrm{Dose}}}}}}_{{{{{{\mathrm{PO}}}}}}}} \right)/\left( {{{{{{\mathrm{AUC}}}}}}_{0 - \infty ,\,{{{{{\mathrm{IV}}}}}}}/{{{{{\mathrm{Dose}}}}}}_{{{{{{\mathrm{IV}}}}}}}} \right)$$

Mean AN15368 plasma concentrations and PK parameters after a single IV or PO dose, and mean AN15368 (total and free) plasma concentrations and PK parameters in the pre- and end-regimen periods are depicted in Supplementary Fig. 4.

### NHP treatment study

A total of 22 rhesus macaques that had been confirmed to be serologically and PCR-positive for *T. cruzi* were utilized in these studies. Using 19 animals in the treatment group provided 85% power of detecting 100% efficacy. The 19 animals were treated with a 30 mg kg^−1^ dose of AN15368 delivered in food treats once a day for 60 d. The remaining 3 animals in the study were maintained as untreated control animals and received food treats but were not dosed with AN15368.

The selected dose of 30 mg kg^−1^ in NHP was determined on the basis of the following rationale. The minimal efficacious dose in mice was determined to be 2.5 mg kg^−1^ (Fig. 3e) and PK studies in mice at a 10 mg kg^−1^ dose yielded an exposure of 17.5 (AUC_0–last_ (μg h kg^−1^); Supplementary Table 6). Assuming linear exposure, a minimal curative exposure in mice was estimated at 4.375 μg h kg^−1^ (that is, 17.5/4). PK analysis of a 30 mg kg^−1^ dose in NHP indicated an exposure of 3.5–4.7 μg h kg^−1^ (Supplementary Fig. 5). On the basis of allometric scaling^15^, the no-observed-adverse-effect level (NOAEL) of 120 mg kg^−1^ in rats translates to a NOAEL dose of 60.5 mg kg^−1^ in monkeys and 19.4 mg kg^−1^ in humans. Thus, the 30 mg kg^−1^ NHP dose was expected to achieve an efficacious level on the basis of PK comparisons of mouse and NHP, and be safe as it is well below the estimated NOAEL dose determined in rats.

Under light anaesthesia/sedation, peripheral blood samples were collected from each animal before treatment and at 7 time points after treatment. Blood analysis (complete blood count, CBC and serum chemistry assays) and physical exams to evaluate the health of the study animals were performed on each animal before the beginning of the study and also at 3 time points during the treatment protocol (see Supplementary Data). At the termination of the study, 9 treated and 2 control animals were euthanized, necropsied, and blood and tissues were evaluated histologically, and using PCR and haemoculture for evidence of active *T. cruzi* infection. The remaining 10 treated and 1 control animals from the study were returned to the breeding colony at the Keeling Center.

### NHP blood and tissue PCR for *T. cruzi* DNA and haemoculture

Blood samples from each macaque were collected at various time points and processed for quantification of *T. cruzi* DNA by real-time qPCR. Between 8 to 10 ml of whole blood collected in EDTA anticoagulant tubes was subjected to DNA extraction using the Omega E.Z.N.A. blood DNA maxi kit (Omega BioTek) following the manufacturer’s instructions for up to 10 ml whole blood and using a total of 650 µl of elution buffer. Each round of extractions included a negative (no-template) control composed of 10 ml PBS. The concentration of DNA in the eluted solution was quantified after each extraction using an Epoch microplate spectrophotometer (BioTek).

DNA from each sample was then subjected to a series of two qPCR assays for detection of *T. cruzi* satellite DNA. The first qPCR used the cruzi 1, 2 primer set and cruzi 3 *Taq*Man probe as previously described^53,54^, using Bio-Rad i*Taq* Universal Probes Supermix (Bio-Rad). This qPCR amplifies a 166 bp region of a repetitive satellite DNA sequence and is sensitive and specific for *T. cruzi* when compared with other PCR techniques^55^. To rule out false negative PCR results due to inhibition, an internal amplification control (IAC) was added to the second qPCR reaction, which was run as a multiplex as previously described, with the cruzi 1/2/3 primers and probe, and the IAC primers and probe^54^, except that the IAC sequence was synthesized as a gene fragment by a commercial laboratory (gBlocks Gene Fragments, Integrated DNA Technologies) and was added at the time of PCR rather than before extraction. Positive (DNA extracted from *T. cruzi* Sylvio X10 clone 4, American Type Culture Collection (ATCC), 50800, known concentration 1.7 × 10^−3^ parasite equivalents) and negative (water) controls were included in each PCR plate for both assays. A C1000 Touch Bio-Rad CFX96 real-time PCR detection system was used for both assays under the following cycling conditions: (1) initial denaturation, 95 °C, 3 min; (2) denaturation, 95 °C, 15 s; (3) annealing, 58 °C, 1 min; (4) ×50 cycles. We selected FAM and VIC channels for each read.

Frozen tissues were screened for *T. cruzi* DNA using 8 mm biopsies (Sklar instruments, 96-1130), collecting 3 to 10 individual ~100 µl tissue punches for each tissue type and one or more pooled samples consisting of five punches from different areas of the tissue, totalling ~500 µl per pool. The tissues sampled included liver, heart, fat, oesophagus, quadricep, bicep, large intestine, and brain, as well as tongue and spleen in a few instances. Two individual and one pooled sample of tissues from an uninfected macaque was collected for each sampling batch. DNA from macaque tissue was extracted and analysed as previously described^32^, with the exceptions that the purification was scaled up to accommodate the larger amount of tissue in the pooled samples and the range for the standards was 2.6 × 10^2^ – 2.6 × 10^−3^ parasite equivalents using the kDNA minicircle S35 and S36 primers. The Bio-Rad CFX manager software version 3.1 was used to analyse PCR data. For samples to be considered positive, both replicates per sample must show a product (cQ value) of <40 and less than that of the included naïve sample, and melt curves had to be in the same temperature range as the standards for each plate.

For haemoculture determinations, peripheral blood from macaques was collected and cultured at 26 °C in supplemented liver digest neutralized tryptose medium as described previously^17^. The presence of *T. cruzi* parasites was assessed every week for 3 months under an inverted microscope. *T. cruzi* DTU of the macaque isolates was determined as previously described^17^.

### Multiplex serological analysis

Luminex-based multiplex serological assays were performed as previously described^16,17^. For a number of smaller proteins, fusions of up to 2 individual genes are employed for some target proteins to expand the array of antibodies being detected while controlling costs and complexity of the assay (TritrypDb.org identifiers: Tc1, fusion of TcBrA4_0116860 and TcYC6_0028190; Tc2, fusion of TcBrA4_0088420 and TcBrA4_0101960; Tc3, fusion of TcBrA4_0104680 and TcBrA4_0101980; Tc4, fusion of TcBrA4_0028480 and TcBrA4_0088260; Tc5, fusion of TcYC6_0100010 and TcBrA4_0074300; Tc7, fusion of TcYC6_0083710 and TcBrA4_0130080; Tc8, TcYC6_0037170; Tc11, TcYC6_0124160; Tc17, fusion of TcBrA4_0028230 and TcBrA4_0029760; Kn107, TcCLB.508355.250; G10, TcCLB.504199.20). Macaque antibody binding to individual beads in the multiplex assays was detected with donkey anti-human IgG (H+L) conjugated to phycoerythrin (Jackson ImmunoResearch) in a 1:200 dilution.

### Statistics and reproducibility

The non-parametric Mann-Whitney U test and the unpaired *t*-test from the software GraphPad Prism v9.4.0 were used. Values are expressed as mean ± s.e.m. Statistical significance was evaluated using *P ≤ 0.05, **P ≤ 0.01 and ***P ≤ 0.001. All mouse experiments were performed at least twice with similar results. All in vitro parasite proliferation assays were repeated at least once with similar results. PCR and western blot assays depicted as representative microphotographs in Fig. 2b were repeated three times and one time, respectively, with similar results. The quantitative liquid chromatography tandem mass spectrometry assay described in Fig. 2d was performed once. Due to cost and complexity, the NHP trial was performed once.

### Reporting summary

Further information on research design is available in the Nature Research Reporting Summary linked to this article.

## Supplementary information

Supplementary Information Supplementary Text, Extended Data figure legends, Supplementary Figs. 1–5, Tables 1–8, Data 1 caption and Fig. 1 source data image.

Reporting Summary

Peer Review File

Supplementary Data 1 NHP haematology, blood chemistry, weight and histology.

## Data Availability

With the exception of mRNASeq data available in the NCBI Sequence Read Archive (SRA; http://www.ncbi.nlm.nih.gov/sra/ under accession numbers SRX13363525–SRX13363532), all data are available in the main text or the Supplementary Information. The CL Brener genome is available at TritrypDB (release-33; https://tritrypdb.org/tritrypdb/app) and the African green monkey genome at the DNA Data Bank of Japan (DDBJ, http://www.ddbj.nig.ac.jp; accession number DRA002256). Source data are provided with this paper.

## Source data

Source Data Fig. 1 Numerical source data.
Source Data Fig. 2 Numerical source data.
Source Data Fig. 3 Numerical source data.
Source Data Fig. 4 Numerical source data.
Source Data Fig. 5 Numerical source data.
Source Data Fig. 2 Unprocessed gels.
Source Data Extended Data Fig. 3 Numerical source data.
Source Data Extended Data Fig. 4 Numerical source data.
