# Supplementary figures and images for: Discovery of an Orally Active Benzoxaborole Prodrug Effective in the Treatment of Chagas Disease in Non-human Primates
Source: Nat Microbiol. Author manuscript; Available in PMC 2022 Oct 3. (PMC9519446; doi:10.1038/s41564-022-01211-y)

Image source data\_Figure 2b. Left panel

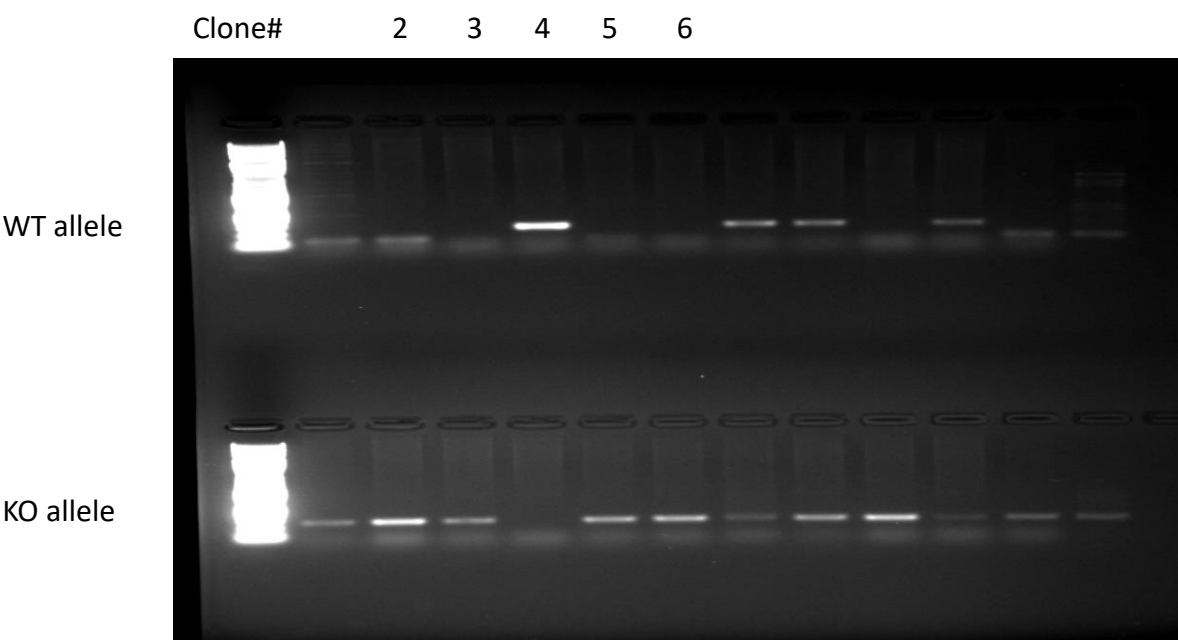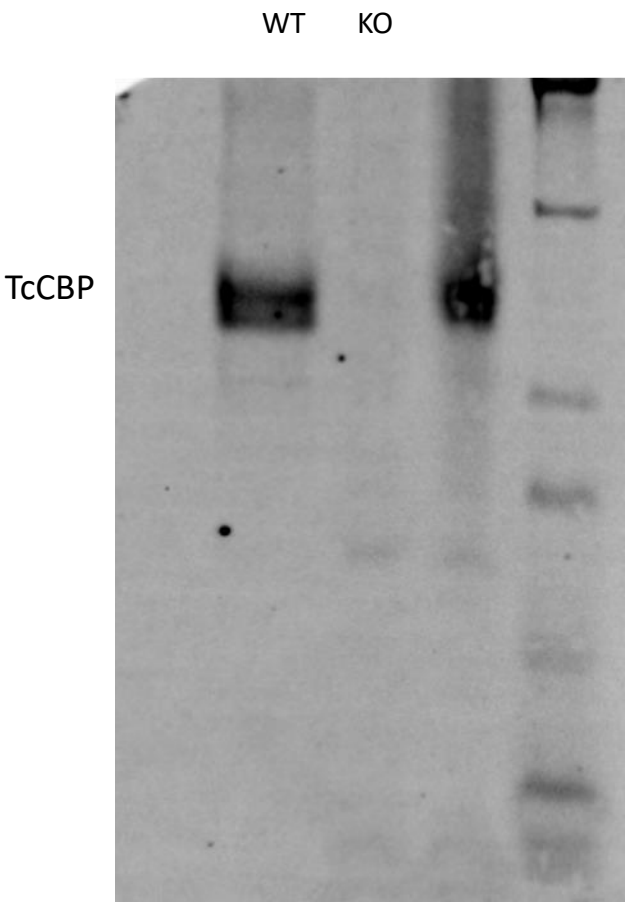

Image source data\_Figure 2b. right panel

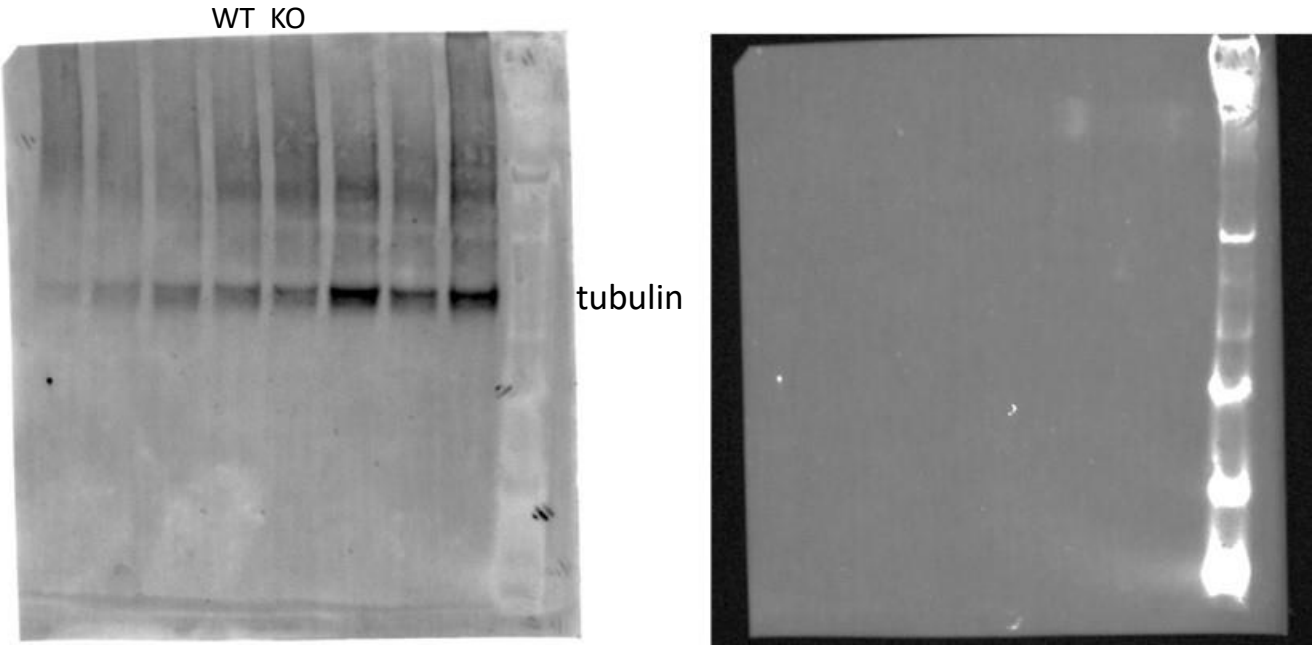

Supplement: Unmodified_gels_Fig2 [file EMS151388-supplement-Unmodified_gels_Fig2.pdf]
