# Supplementary material for: Discovery of an orally active benzoxaborole prodrug effective in the treatment of Chagas disease in non-human primates
Source: Nat Microbiol. 2022 Sep 5;7(10):1536–46. doi: 10.1038/s41564-022-01211-y (PMC9519446; doi:10.1038/s41564-022-01211-y)

nature portfolio

https://doi.org/10.1038/s41564-022-01211-y

Supplementary information

Discovery of an orally active benzoxaborole prodrug effective in the treatment of Chagas disease in non-human primates

In the format provided by the authors and unedited

Supplementary Information for

Discovery of an Orally Active Benzoxaborole prodrug effective in the Treatment of Chagas Disease in non-human primates

Angel M. Padilla[1†], Wei Wang[1†], Tsutomu Akama[2], David S. Carter[2], Eric Easom[2], Yvonne Freund[2], Jason S. Halladay[2], Yang Liu[2] Sarah A. Hamer[3], Carolyn L. Hodo[3], Gregory K. Wilkerson[4], Dylan Orr[1], Brooke White[1], Arlene George[1], Huifeng Shen[1], Yiru Jin[5], Michael Zhou Wang[5], Susanna Tse[6], Robert T. Jacobs[2‡], Rick L. Tarleton[1‡*]

Correspondence to: tarleton@uga.edu

This file includes:

Supplemental Text
Extended data legends
Figs. S1 to S5
Tables S1 to S8
Captions for Data S1
Source Data image for Fig. S1

Other Supplementary Materials for this manuscript include the following:

Data S1
- Hematology
- Blood Chemistry
- Weight
- Histology

Supplementary Text

Compound Synthesis

All compounds described in this paper were prepared as described in US Patent 10,882,272, granted January 5, 2021.

Summarized below are the syntheses of AN14353 and AN15368 that are representative of those described in this patent.

Preparation of 1-hydroxy-7-methyl-1,3-dihydrobenzo[c][1,2]oxaborole-6-carboxylic acid 7.

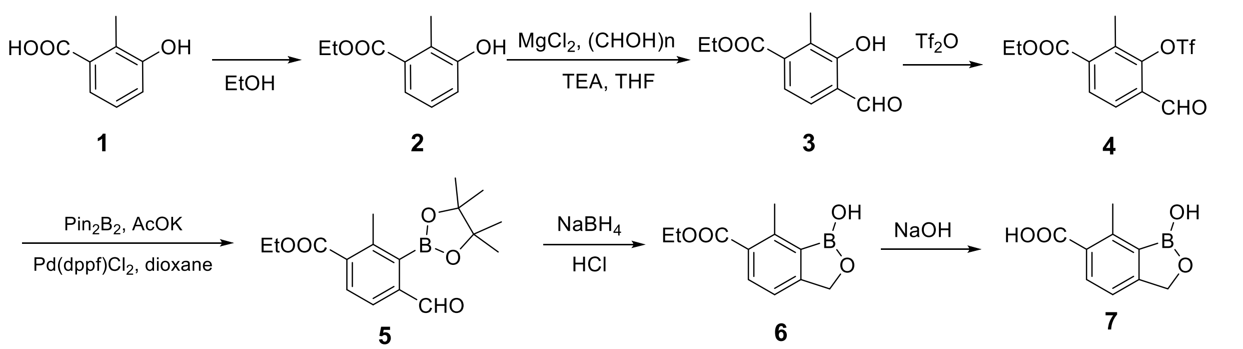

a. **Ethyl 3-hydroxy-2-methylbenzoate 2**. To a solution of **1** (1.65 kg, 10.8 mol) in EtOH (6.50 L) was added con. H_2SO_4 (326 g, 3.25 mol). The reaction mixture was heated 105 °C for 24 h. TLC showed **1** was consumed completely. The mixture was cooled to 15 °C and concentrated to give the crude product. The residue was poured into 2 M $NaHCO_3$ (aq., 3 L) and the solid was filtered. The filtrate was concentrated to give **2** (1.75 kg, 90%) as brown solid.

   ^{1}H NMR (400 MHz, $CDCl_3$) δ 7.41 (d, J = 7.9 Hz, 1H), 7.11 (t, J = 7.9 Hz, 1H), 6.94 (d, J = 7.9 Hz, 1H), 4.58 (br. s., 1H), 4.37 (q, J = 7.4 Hz, 2H), 2.46 (s, 3H), 1.40 (t, J = 7.1 Hz, 3H).

b. **Ethyl 4-formyl-3-hydroxy-2-methylbenzoate 3**. To a solution of **2** (800 g, 4.44 mol) in THF (6.50 L) were added $MgCl_2$ (634 g, 6.66 mol, 273 mL), TEA (1.80 kg, 17.8 mol) and (HCHO)n (600 g, 6.66 mol). The mixture was immediately heated to 90 °C for 14 h. TLC showed the **2** was consumed completely. The reaction mixture was cooled to 15 °C, added ice H_2O (3 L) and slowly added 12 M HCl (1.5 L). The mixture was stirred half an hour and then extracted with EtOAc (2 L). The combined organic layer was washed by sat. $NaHCO_3$ to neutral, dried over Na_2SO_4, filtered and concentrated under reduced

pressure to give **3** (880 g, crude) as brown oil.

^{1}H NMR (400 MHz, $CDCl_3$) δ 11.40 (s, 1H), 9.93 (s, 1H), 7.46 (d, *J* = 7.6 Hz, 1H), 7.37 (d, *J* = 8.0 Hz, 1H), 4.40 (q, *J* = 7.4 Hz, 2H), 2.44 (s, 3H), 1.41 (t, *J* = 7.1 Hz, 3H).

c. **Ethyl 4-formyl-2-methyl-3-(((trifluoromethyl)sulfonyl)oxy)benzoate 4**. To a solution of **3** (900 g, 4.32 mol) in DCM (7.56 L) was added pyridine (1.02 kg, 12.9 mol) and DMAP (27 g, 221 mmol) respectively. The mixture was cooled to 0 °C and Tf_2O (1.60 kg, 5.66 mol) was added drop wise. The reaction mixture was warmed to 15 °C and stirred for 1 h. TLC showed **3** was consumed completely. The mixture was quenched by water (7.65 L) and then extracted with DCM (7.65 L x 2). The combined organic layer was washed with water (2 L), dried over Na_2SO_4, filtered and concentrated under reduced pressure to give **4** (685 g, 47%) as a light yellow oil.

^{1}H NMR (400 MHz, $CDCl_3$) δ 10.27 (s, 1H), 7.99 (d, *J* = 8.0 Hz, 1H), 7.91-7.87 (m, 1H), 4.43 (q, *J* = 7.0 Hz, 2H), 2.64 (s, 3H), 1.43 (t, *J* = 7.3 Hz, 3H).

d. **Ethyl 4-formyl-2-methyl-3-(4,4,5,5-tetramethyl-1,3,2-dioxaborolan-2-yl)benzoate 5**. To a solution of **4** (1.00 kg, 2.94 mol), bis(pinacolato)diboron (1.12 kg, 4.41 mol) and KOAc (573 g, 5.84 mol) in 1,4-dioxane (6.50 L) was added Pd(dppf)Cl_2.CH_2Cl_2 (150 g, 184 mmol). The mixture was heated at 85 °C for 15 h under N_2 atmosphere. TLC showed **4** was consumed completely. The mixture was cooled to 15 °C, filtered and concentrated to give the crude product. The residue was purified by column chromatography (SiO_2, Petroleum ether/Ethyl acetate = 40/1 to 4:1) to give **5** (942 g, crude) as a yellow oil.

e. **Ethyl 1-hydroxy-7-methyl-1,3-dihydrobenzo[c][1,2]oxaborole-6-carboxylate 6**. To a solution of **5** (1.20 kg, 3.77 mol) in MeOH (300 mL) and THF (6.00 L) was added $NaBH_4$ (80 g, 2.11 mol) in portions at 0 °C. Then the reaction mixture was stirred at 15 °C for 1 h. HPLC showed **5** was consumed completely. The reaction solution was adjusted to pH = 4 with 2 M HCl and then the organic layer removed in vacuo. The mixture was filtered. The cake was washed with Petroleum ether (5 L) and dried in vacuum to give **6** (665 g, 80%) as a white solid.

^{1}H NMR (400 MHz, DMSO-d_6) δ 9.18 (s, 1H), 7.89 (d, *J*=8.0 Hz, 1H), 7.32 (d, *J*=8.0 Hz, 1H), 5.00 (s, 2H), 4.30 (q, *J*=7.0 Hz, 2H), 2.68 (s, 3H), 1.33 (t, *J*=7.0 Hz, 3H).

f. **1-Hydroxy-7-methyl-1,3-dihydrobenzo[c][1,2]oxaborole-6-carboxylic acid 7**. To a

mixture of **6** (867 g, 3.94 mol) in H_2O (5.00 L) was added NaOH (394 g, 9.85 mol) in one portion. The solution was heated at 40 °C for 3 hours. HPLC showed **6** was consumed completely. This batch was work-up together with the other batches acidified with 2 M HCl to pH = 2. The solid was filtered and washed with H_2O (10 L). The cake was dried to give **7** (2.00 kg, 87%) as a white solid.

^{1}H NMR (400 MHz, DMSO-d_6) δ 9.13 (br. s., 1H), 7.89 (d, J = 8.0 Hz, 1H), 7.28 (d, J = 8.0 Hz, 1H), 4.98 (s, 2H), 2.68 (s, 3H).

Synthesis of 3,4-Difluorobenzyl(1-hydroxy-7-methyl-1,3-dihydrobenzo[c][1,2]oxaborole -6-carbonyl)-L-valinate (AN14353)

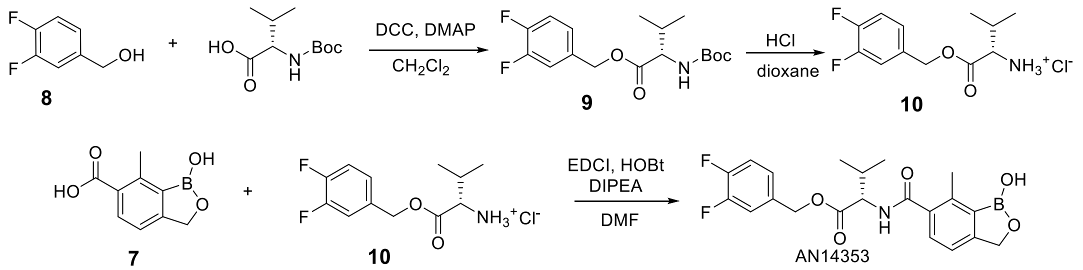

a. **3,4-Difluorobenzyl (tert-butoxycarbonyl)-L-valinate 9**. To a solution of N-BOC-(S)-valine (2.6 g, 12.15 mmol, 1.00 eq) and 3,4-difluorobenzylalcohol (2.8 g, 19.44 mmol, 248.10 mL) in dry DCM (65 mL) was added DCC (4.45 g, 21.56 mmol, 838 mL) and DMAP (0.219 g, 1.797 mmol). The reaction mixture was stirred at 25°C for 18 h. The mixture was filtered and washed with DCM (100 mL) and concentrated to give the crude product. The residue was purified via column chromatography (SiO2, Petroleum ether/Ethyl acetate = 50/1 to 10:1) to give 3,4-difluorobenzyl (tert-butoxycarbonyl)-L-valinate **9** (3.7 g, 88% yield) as a yellow sticky solid.

b. **3,4-Difluorobenzyl L-valinate hydrochloride 10**. To a stirred solution of **9** (5 g, 14.57 mmol) in dioxane (25 mL) was added 3N HCl/dioxane (25 mL). The reaction mixture was stirred at 25 °C for 18 h. The solvent was removed under reduced pressure and triturated with diethyl ether to give **10** (2.65 g, 63%) as a white solid.

c. **3,4-Difluorobenzyl(1-hydroxy-7-methyl-1,3-dihydrobenzo[c][1,2]oxaborole -6-carbonyl)-L-valinate** A mixture of carboxylic acid **7** (0.7 g, 3.64 mmol), amine **10** (1.06 g, 4.37 mmol) and DIPEA (2.01 mL, 10.93 mmol) in DMF (20 mL) was added EDCI (1.04 g, 5.47 mmol) and HOBt (738 mg, 5.47). The mixture was stirred at RT for 18hrs. The crude product was purified by reversed phase chromatography to get AN14353 (350

mg, 23% yield) as a white solid. 1H NMR (400 MHz, DMSO-d6) δ 9.02 (s, 1H), 8.58 (d, J = 7.94 Hz, 1H), 7.54 - 7.39 (m, 2H), 7.35 - 7.17 (m, 3H), 5.15 (s, 2H), 4.95 (s, 2H), 4.33 (t, J = 7.1 Hz, 1H), 2.41 (s, 3H), 2.20 - 2.08 (m, 1H), 0.94 (d, J = 6.6 Hz, 3H), 0.92 (d, J = 6.6 Hz, 3H); ESIMS m/z 418 [M+H]+; HPLC purity: 98.35% (220 nm), 98.15% (254 nm).

Synthesis of (Tetrahydro-2H-pyran-4-yl)methyl (1-hydroxy-7-methyl-1,3-dihydrobenzo[c][1,2]oxaborole-6-carbonyl)-L-valinate (AN15368)

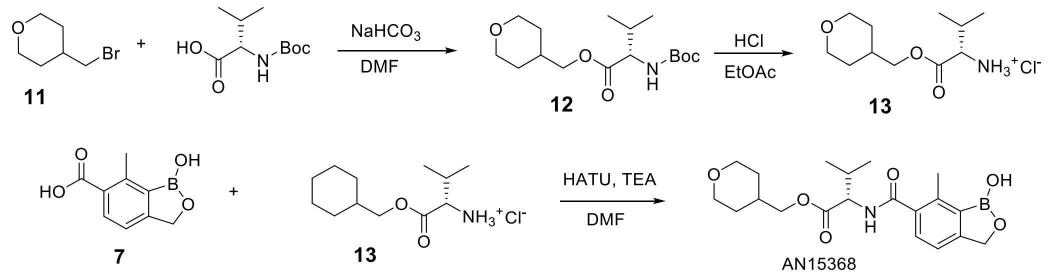

a. **(Tetrahydro-2H-pyran-4-yl)methyl (tert-butoxycarbonyl)-L-valinate 12**. A mixture of (tert-butoxycarbonyl)-L-valine (3.64 g, 16.75 mmol), 4-(bromomethyl)tetrahydro-2H-pyran (3.00 g, 16.75 mmol) and $NaHCO_3$ (2.81 g, 33.50 mmol) in DMF (30 mL) was stirred at 70 °C for 12 hours under N_2 atmosphere. The reaction mixture was diluted with H_2O (100 mL) and extracted with MTBE (50 mL x 2). The combined organic layers were washed with brine (20 mL x 2), dried over Na_2SO_4, filtered and concentrated under reduced pressure to give (tetrahydro-2H-pyran-4-yl)methyl (tert-butoxycarbonyl)-L-valinate (5 g, 95%) as a pale yellow oil) which was used into the next step without further purification. ^{1}H NMR (400 MHz, $CDCl_3$) δ 5.01 (d, J = 8.4 Hz, 1H), 4.22 (dd, J = 8.8 Hz, 4.8 Hz, 1H), 4.00-3.97 (m, 4H), 3.40 (t, J = 11.2 Hz, 2H), 2.16-2.11 (m, 1H), 1.96-1.90 (m, 1H), 1.63 (d, J = 13.2 Hz, 2H), 1.45 (s, 9H), 0.97 (d, J = 6.4 Hz, 3H), 0.90 (d, J = 7.2 Hz, 3H).

b. **(Tetrahydro-2H-pyran-4-yl)methyl L-valinate hydrochloride 13**. To a solution of (tetrahydro-2H-pyran-4-yl)methyl (tert-butoxycarbonyl)-Lvalinate (5.00 g, 15.85 mmol) in EtOAc (50 mL) was added HCl/EtOAc (6 M, 26.42 mL). The mixture was stirred at 15 °C for 2 hours, then was concentrated under reduced pressure to give (tetrahydro-2H-pyran-4-yl)methyl L-valinate hydrochloride (3.80 g, yield 95%) as a white solid. ^{1}H NMR (400 MHz, DMSO-d6) δ 8.53 (br. s., 2H), 4.04 (d, J = 6.0 Hz, 2H), 3.88-3.84 (m, 3H), 3.29 (t, J = 11.2 Hz, 2H), 2.21-2.17 (m, 1H), 1.91-1.86 (m, 1H), 1.59 (d, J = 13.65

Hz, 2H), 1.29-1.34 (m, 2H), 0.97 (dd, J = 16.4, 7.2 Hz, 6H).

c. A mixture of carboxylic acid 7 (2.00 g, 10.42 mmol), TEA (3.16 g, 31.26 mmol) and HATU (4.75 g, 12.50 mmol) in DMF (10 mL) was degassed and purged with N_2 for 3 times and stirred at 15°C for 10 mins. Amine **13** (2.75 g, 10.94 mmol) was added to the reaction mixture and stirred at 15 °C for 20 mins under N_2 atmosphere. After being filtered, the mixture was purified by prep-HPLC (column: Phenomenex Synergi Max-RP 250*80 10u; liquid phase: [A-TFA/H2O = 0.075% v/v; B-ACN] B%: 10%- 40%, 20 mins]) to give (tetrahydro-2H-pyran-4-yl)methyl (1-hydroxy-7-methyl-1,3-dihydrobenzo[c][1,2]oxaborole-6-carbonyl)-L-valinate **AN15368** (1.300 g, 25%) as a white solid. ^{1}H NMR (400 MHz, DMSO-d6) δ 9.04 (br s, 1H), 8.54 (d, J = 7.6 Hz, 1 H), 7.37 (d, J = 7.6 Hz, 1H), 7.24 (d, J = 7.6 Hz, 1H), 4.97 (s, 2H), 4.31 (t, J = 7.0 Hz, 1H), 3.96 (d, J = 6.0 Hz, 2H), 3.85 (d, J = 9.2 Hz, 2H), 3.29 (t, J = 11.6 Hz, 2H), 2.47 (s, 3H), 2.14 (dd, J = 13.2, 6.4 Hz, 1H), 1.87 (br s, 1H), 1.59 (d, J = 12.0 Hz, 2H), 1.31-1.23 (m, 2H), 0.96 (d, J = 4.4 Hz, 6H); ESI-MS m/z 390 [M+H]+; HPLC purity: 98.49% (220 nm), 89.53% (254 nm).

Figure legends – Extended data

Extended data 1: Structure activity relationships of benzoxaboroles and their activity on different genetic types of *T. cruzi*. a. Substitutions in the benzoxaborole ring C(7) position of AN10443 strongly affects in vitro activity against *T. cruzi,* with larger substitutions decreasing activity. **b.** *T. cruzi* of diverse genetic types (DTUs) are susceptible to AN14353-mediated killing (n=3 biological replicates for each profile determination)

Extended data 2. Pre-, and post-treatment infection status of NHP. Euth/Hem = hemoculture results of blood collected at time of euthanasia. LTF = lost to follow-up due to the animal's death from traumatic lesions it acquired through conspecific interactions.

Extended data 3. **Declining IgG levels to recombinant *T. cruzi* proteins over time in AN15368-treated NHP.** Luminex-based pre- and post-treatment IgG responses to recombinant *T. cruzi* proteins in AN15368-treated macaques not pictured in Fig. 4 (see Methods for identification of recombinant proteins).

Extended data 4. AN15368 is activated by a *T. cruzi* serine carboxypeptidase and targets CPSF3. a. Fold change in CPSF3 transcripts by qRT-PCR in two CPSF3-overexpressing *T. cruzi* lines (n=3 biological replicates). **b.** Overexpression of CPSF3 in *T. cruzi* results in increased resistance to all AN15368 analogues (CSPF3-OE: n=3 biological replicates; WT: n=2 biological replicates). **c.** AN15368 analogues require activation by the *T. cruzi* CBP in order to efficiently kill intracellular *T. cruzi* as demonstrated by the increased

resistance of CBP deficient parasites (TcCBP KO: n=3 biological replicates; WT: n=2 biological replicates). **d.** AN15368 analogues with poor activity against intracellular *T. cruzi* amastigotes have low nanomolar activity on extracellular amastigotes (n=3 biological replicates). Data are presented as mean values +/- SD.

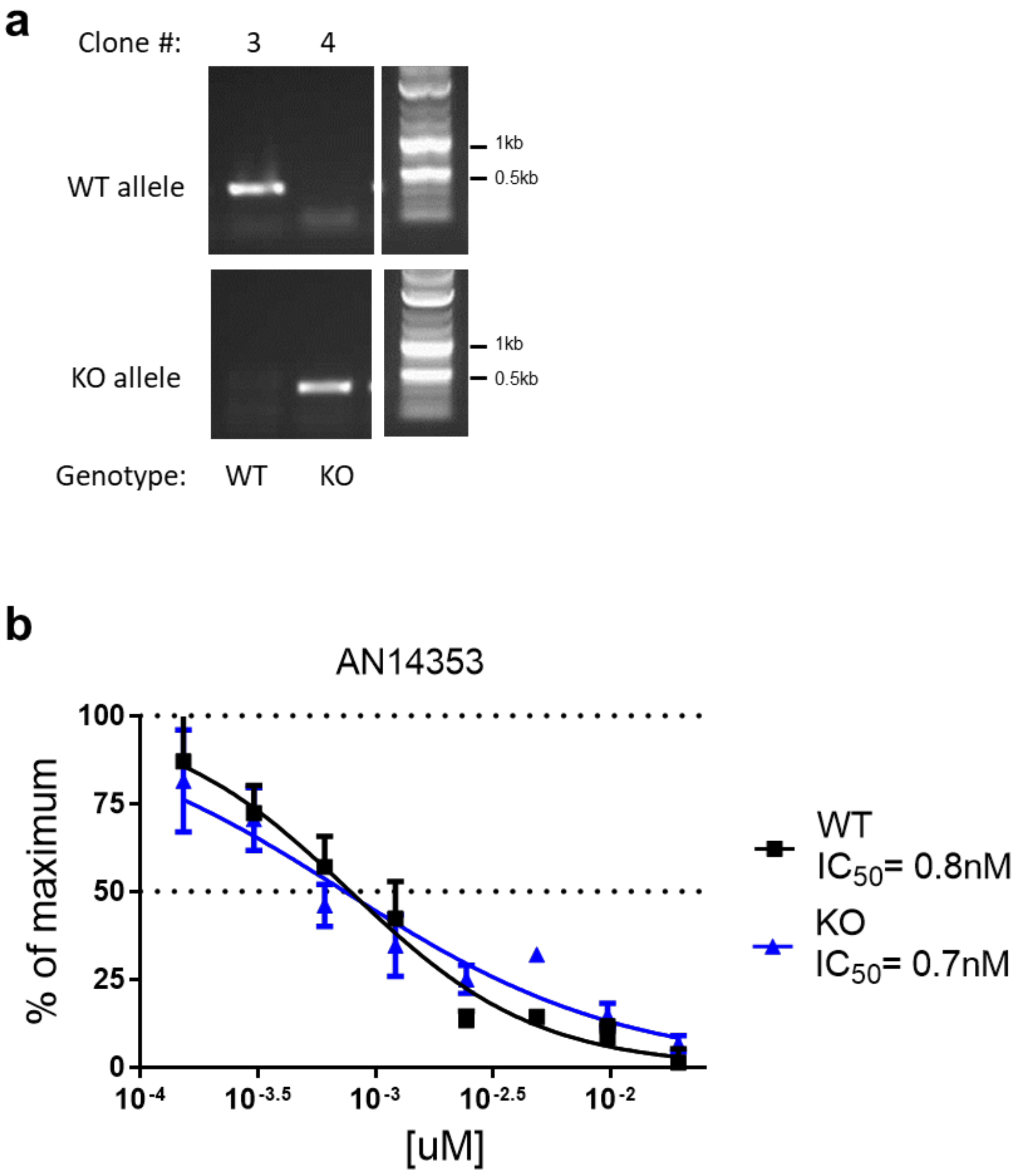

Fig. S1. Lack of impact of metallocarboxypeptidase 2 (TcCLB.504045.60) disruption (**a**) on activity of AN14353 on intracellular amastigote (**b**). Representative microphotographs of assays repeated at least three times are shown. Data are presented as mean values +/- SEM; n= 3 biological replicates.

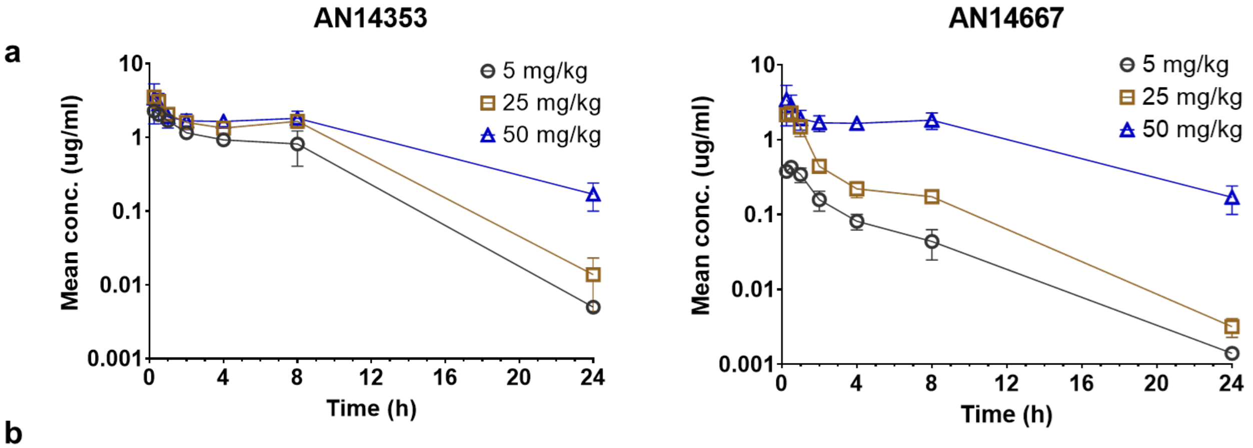

b

	AN14353				AN14667	Ratio AN14667/AN14353 AUC
dose	Cmax (µg/mL)	Fold Change from 5 mg/kg	AUC0-last (µg•hr/mL)	Fold Change from 5 mg/kg	AUC0-last (µg•hr/mL)	
5	2.28	1	15.3	1	1.05	0.07
25	3.53	1.5	18.8	1.22	4.71	0.25
50	3.43	1.5	24.0	1.58	6.68	0.28

Fig. S2. Pharmacokinetics of AN14353 and its carboxylic acid product AN14667 in mice. (**a**) Time vs. concentration curves of substrate and product following oral administration of AN14353 (Data are presented as mean values +/- SEM; n= 3 animals), and (**b**) lack of dose proportionality of exposure with AN14353.

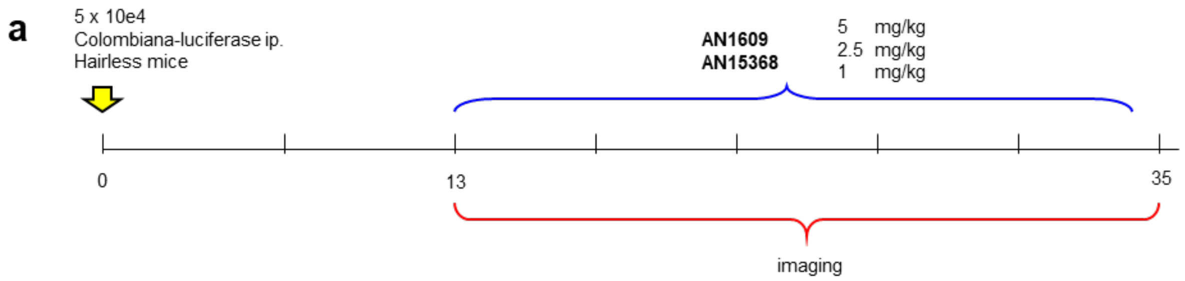

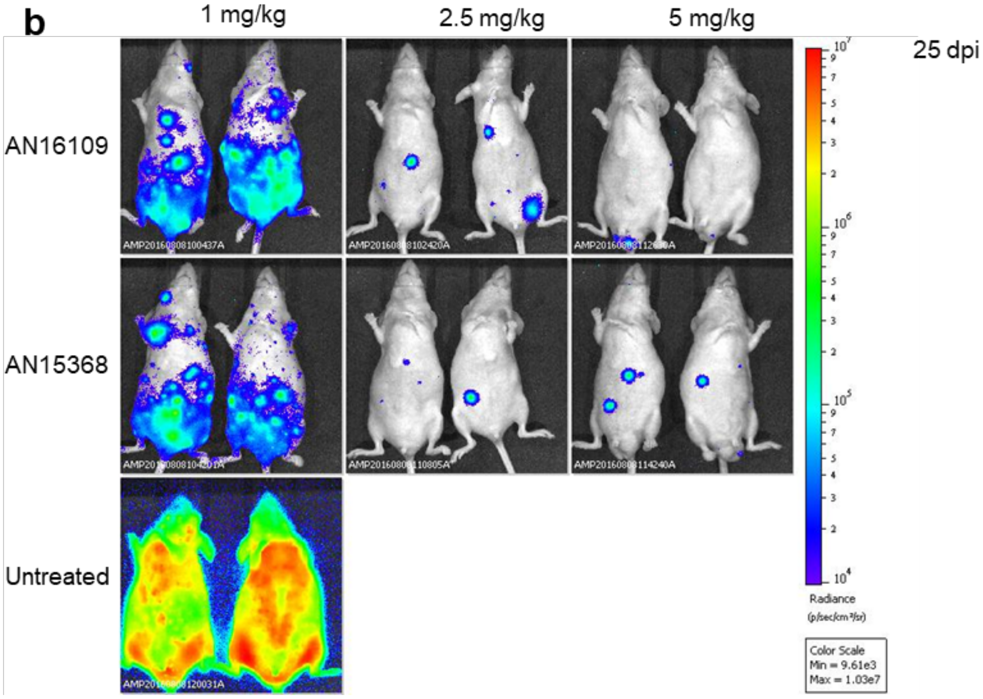

Fig. S3. Short-term (non-cure) treatment course. **a**, Experiment schedule for assessment of low dose treatment. On days 13-33 post infection, oral doses of AN15368 or AN16109 were administered at the indicated concentrations to hairless mice infected i.p. with $5x10^4$ Luciferase-expressing *T. cruzi*. **b**, Representative whole mouse images of bioluminiscence signals acquired and quantified throughout the treatment by in vivo imaging. (n=4-7 mice).

a

Rat IV (2 mg/kg)		Rat PO (10 mg/kg)	
Cmax (µg/mL)	1.70 ± 0.2	Cmax (µg/mL)	2.10 ± 0.29
Cl (mL/h/kg)	2258	AUC0-last (hr*µg/mL)	3.34
Vss (mL/kg)	1115	%F	76.1
AUC0-last (hr*µg/mL)	0.883		

b

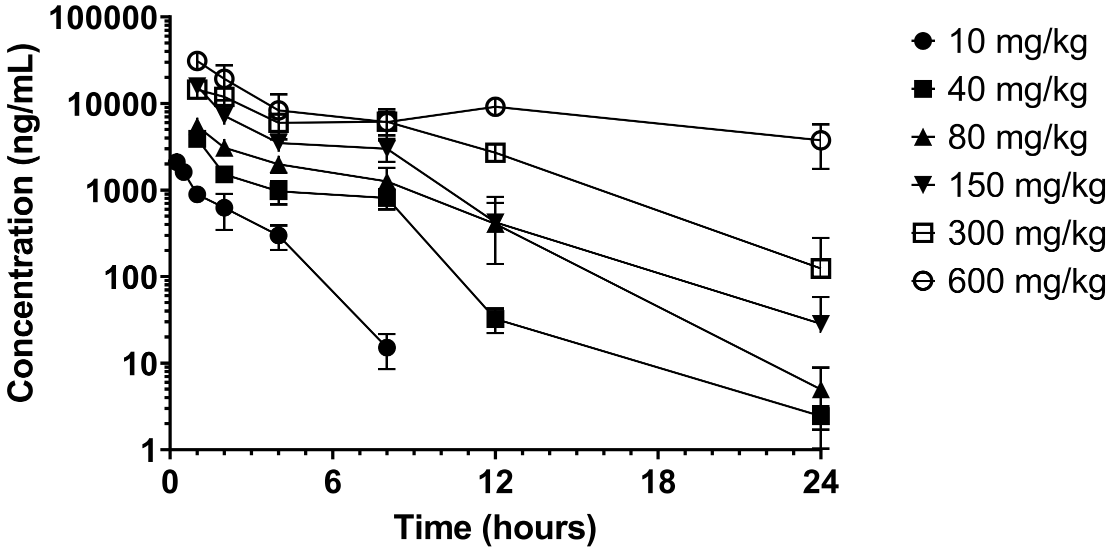

Fig. S4. a, Pharmacokinetics for AN15368 in rats and **b,** evidence for dose proportional exposure for AN15368 in rats. Data are presented as mean values +/- SEM; n= 3 animals.

a

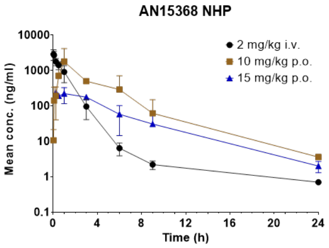

	IV, 2 mg/kg	PO, 10 mg/kg	PO, 15 mg/kg
C_{max} (ng/mL)	NA	1922 ± 2219	262 ± 70.2
t_{max} (hour)	NA	3.3 ± 2.5	0.5 ± 0.4
AUC_{0-24} (ng*h/mL)	2784 ± 1066	5239 ± 3133	1317 ± 416
$AUC_{0-\infty}$ (ng*h/mL)	2791 ± 1063	5262 ± 3133	1329 ± 418
$t_{1/2}$ (hour)	6.7 ± 1.5	4.6 ± 1.5	4.2 ± 1.5
CL_p (mL/min/kg)	13.0 ± 4.3	NA	NA
%F	NA	38.5 ± 6.5	6.5 ± 0.5

b

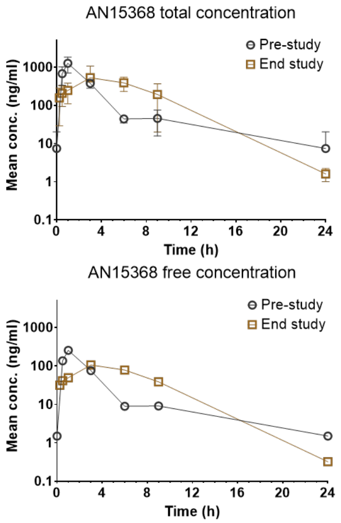

	Pre-treatment			Post-treatment
	30mg/kg PB	50 mg/kg PM	50 mg/kg PM	Day 60
Total Cmax (ng/mL)	639±477	1279±1146	1962±1397	1272±564
Free Cmax (ng/mL)	128±95	256±229	392±279	254±113
tmax (h)	5.0±1.7	3.2±2.8	4.0±1.7	1*
Total AUC0-24 (ng*h/mL)	4665±1579	7308±4568	11450±4539	3477*
Free AUC0-24 (ng*h/mL)	933±316	1462±914	2290±908	695*

Note: * parameter derived from mean plasma concentration vs. time profile on Day 60

Fig. S5. a. Plasma concentrations and pharmacokinetics of AN15368 after a single IV (2 mg/kg) or oral (10 mg/kg in CMC/Tween; 15 mg/kg admixed in food) in rhesus macaques. **b.** Pharmacokinetics of AN15368 in rhesus macaques prior to beginning of daily dosing (pre-study) and on the 60th day of dosing (end study) after a single dose (Pre-treatment, Periods 1, 2 and 3) or multiple (Post-treatment, Day 60) oral administration of 30 mg/kg/day. Data are presented as mean values +/- SEM; n=3 animals for each period/treatment).

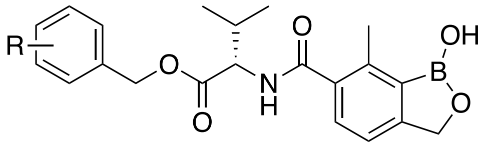

AN #	R	*T. cruzi* IC50 (nM)	mouse S9 Clint (μL/min/mg)	cLogD	Solubility (pH 7.4 PBS, μM)
AN11735	H	4.0	NT	2.70	200
AN11736	4-F	0.9	5.4	2.90	35
AN14335	4-Cl	1.3	16.6	3.30	3.1
AN14336	4-CF3	1.4	NT	3.60	3.1
AN14353	3,4-F_2	6.0	10.2	3.00	25
AN14365	3-Cl	1.0	17.1	3.20	35
AN14393	4-CN	1.6	7.6	2.50	200
AN14416	3-CF_3	1.1	NT	3.60	4.4
AN14429	3-CN	1.0	9.1	2.50	100
AN14500	3-CF_3,4-F	0.7	13.8	3.70	50
AN14502	4-O$(CH_2)_2$pyrrolidine	16.0	1.5	2.60	400
AN14559	4-OCF_3	8.1	10.6	4.40	1.6
AN14560	3-OCF_3	0.7	11.8	4.40	1.6
AN14561	4-SO_2CH_3	126	2.6	1.50	283

Table S1. SAR of Substituted Benzyl Esters in the C(7)-Methylbenzoxaborole Series. n = 3 biological replicates per dilution/determination.

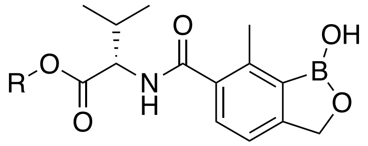

AN #	R	*T. cruzi* IC50 (nM)	mouse S9 Clint (μL/min/mg)	cLogD	Solubility (pH 7.4 PBS, μM)
AN14728	Me	73	1.0	0.9	400
AN15280	iPr	12	1.5	1.7	400
AN15300	iBu	4	3.8	2.2	400
AN15134	tBu	>1250	NT	1.8	200
AN15226	$-(CH_2)_2OCH_3$	19	1.0	0.8	400
AN15129	$-(CH_2)_2$morpholinyl	121	1.4	0.6	400
AN15143	$-(CH_2)_2$pyrrolidinyl	1193	NT	1.1	283
AN15144	$-(CH_2)_2$piperazinyl(NMe)	>1250	NT	0.6	200
AN15192	2-pyridylmethyl	10	7.9	1.6	400
AN15078	3-pyridylmethyl	9	5.2	1.4	400
AN15159	4-pyridylmethyl	30	6.4	1.4	400
AN14504	6-CF_3-3-pyridylmethyl	16	3.5	2.7	200
AN15077	pyrazinylmethyl	25	1.6	1.2	400
AN15410	2-thiazolylmethyl	45	1.4	1.3	400
AN15473	4-thiazolylmethyl	31	1.5	1.2	200
AN15389	5-thiazolylmethyl	27	1.8	1.2	400
AN15658	4-imidazolylmethyl	>1250	6.5	0.6	NT
AN15678	2-imidazolylmethyl	>1250	NT	0.7	NT

Table S2. SAR of Simple Aliphatic and Heterocyclic Esters in the C(7)-Methylbenzoxaborole Series. n = 3 biological replicates per dilution/determination. NT = not tested

	in vitro metabolism		IV @ 2 mg/kg				PO @ 10 mg/kg		
AN #	Mouse S9 Cl_{int} (µL/min/mg)	Human S9 Cl_{int} (µL/min/mg)	C_{max} (µg/mL)	Cl_{plasma} (mL/hr/kg)	Vdss (mL/kg)	$AUC_{0\text{-}last}$ (µg•hr/kg)	C_{max} (µg/mL)	$AUC_{0\text{-}last}$ (µg•hr/kg)	%F
AN11736	5.4	NT	4.5	138	663	14.2	NT	NT	NT
AN14353	10.2	17.8	2.6	437	706	3.2	2.1	12.8	68
AN14504	3.5	6.3	3.9	437	652	5.0	2.9	19.0	75
AN14557	18	23.8	3.1	905	750	2.0	2.1	10.0	100
AN15078	5.2	4.7	NT	NT	NT	NT	3.9	9.6	NC
AN15129	1.4	2.5	5.2	142	305	9.4	6.2	16.4	36
AN15159	6.4	3.1	NT	NT	NT	NT	3.3	5.8	NC
AN15192	7.9	15.8	3.6	1048	629	1.9	2.9	2.6	27
AN15226	1.0	3.4	NT	NT	NT	NT	2.7	8.4	NC

Table S3. Pharmacokinetic Properties of Lead Compounds. n = 3 biological replicates per dilution/determination. NT = not tested; NC = not calculated.

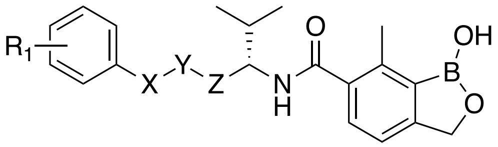

AN #	R1	X-Y-Z	*T. cruzi* IC_{50} (nM)	
AN11735	H	CH_2OCO	ester	4
AN15247	H	CH_2NHCO	amide	>1250
AN14973	4-F	CH_2OCH_2	ether	>1250
AN15158	H	CH_2CH_2CO	ketone	>1250
AN15356	H	SO_2NHCO	acylsulfonamide	>1250
AN14562	4-F	1,2,4-oxadiazole	ester isostere	>1250

Table S4. Ester Replacements in the C(7)-Methylbenzoxaborole Series. n = 3 biological replicates per dilution for each determination.

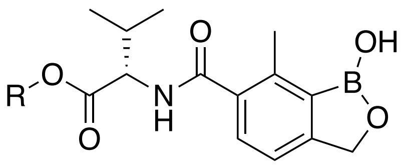

AN #	R	T. cruzi IC_{50} (nM)	mouse S9 Clint (µL/min/mg)	cLogD	Solubility (pH 7.4 PBS, µM)
AN15226	$-(CH_2)_2OCH_3$	19	1.0	0.8	400
AN15368	$-CH_2$(4-tetrahydropyranyl)	5	1.0	1.2	400
AN15572	4-tetrahydropyranyl	11	1.0	0.8	400
AN15573	3-oxetanyl	24	1.0	0.7	200
AN15664	$-CH(CH_2OCH_3)_2$	74	1.0	0.7	NT
AN15666	(S)-3-tetrahydrofuranyl	90	1.0	0.7	NT
AN15667	(R)-3-tetrahydrofuranyl	106	1.0	0.7	NT
AN15828	$-CH_2$(3-oxetanyl)	51	NT	0.5	NT
AN15876	(R)-CH_2(2-tetrahydrofuranyl)	7	16.5	1.2	NT
AN15953	$-CH_2$(4-F-tetrahydropyran-4-yl)	7	2.4	0.7	NT
AN15954	-CH2(2-(1,4-dioxanyl)	15	NT	0.5	NT
AN16108	(S)-CH_2(3-tetrahydrofuranyl)	3.5	NT	0.9	NT
AN16109	(R)-CH_2(3-tetrahydrofuranyl)	5.3	2.4	0.9	NT
AN14817	$-CH_2$(4-fluorophenyl)	12	1.1	1.4	50
AN15170	$-CH_2$(3,4-difluorophenyl)	19	1.5	1.5	100
AN15171	$-CH_2$(3,5-difluorophenyl)	21	1.2	1.5	100
AN15955	$-CH_2$(cyclopentyl)	35	3.4	1.0	NT
AN15988	$-CH_2$(2-pyridyl)	92	NT	0.1	NT
AN16235	$-(CH_2)_2$morpholinyl	>1250	NT	-0.9	NT
AN16236	(R)-CH_2(2-tetrahydrofuranyl)	100	NT	-0.3	NT
AN16330	$-CH_2$(4-tetrahydropyranyl)	302	NT	-0.3	NT

Table S5. SAR of Aliphatic and Cyclic Ether-Containing Esters and Hydroxyvaline Esters in the C(7)-Methylbenzoxaborole Series. n = 3 biological replicates per dilution/determination. NT = not tested.

AN #	in vitro metabolism		Plasma Protein Binding		IV @ 2 mg/kg				PO @ 10 mg/kg		
	Mouse S9 Cl_{int} (µL/min/mg)	Human S9 Cl_{int} (µL/min/mg)	Mouse (% @ 2 µM)	Human (% @ 2 µM)	C_{max} (µg/mL)	Cl_{plasma} (mL/hr/kg)	Vdss (mL/kg)	$AUC_{0\text{-last}}$ (µg•hr/kg)	C_{max} (µg/mL)	$AUC_{0\text{-last}}$ (µg•hr/kg)	%F
AN15226	1.0	3.4	98.7	NT	NT	NT	NT	NT	2.69	8.4	NC
AN15368	1.0	1.5	97.8	73.9	4.47	246	641	8.13	7.06	17.5	43
AN15572	1.0	1.0	92.8	NT	NT	NT	NT	NT	3.99	15.9	NC
AN15876	3.3	16.5	97.3	60.1	NT	NT	NT	NT	4.19	4.3	NC
AN16109	2.4	2.4	96.2	68.9	4.68	221	454	7.81	6.80	17.7	49
AN14817	1.1	1.4	97.3	88.1	1.60	947	2079	1.99	1.41	8.6	83
AN16236	NT	NT	NT	NT	1.65	1068	1489	1.86	3.12	8.4	90

Table S6. Pharmacokinetic Properties of Hydrophilic and Hydroxyvaline Esters. n = 3 biological replicates per dilution/determination.

			Days post-treatment										
Animal ID	Pre-Study 1	Pre-Study 2	0	20	34	54	68	103	131	145	460	945	1281
**Treated**													
T1	28.69	29.33	NoCq	NoCq	NoCq	NoCq	NoCq	NoCq	NoCq	EUTH			
T2	27.34	28.75	NoCq	NoCq	NoCq	NoCq	NoCq	NoCq	NoCq	EUTH			
T3	33.38	NoCq	NoCq	NoCq	NoCq	NoCq	NoCq	NoCq	NoCq	EUTH			
T4	28.87	27.83	NoCq	NoCq	NoCq	NoCq	NoCq	NoCq	NoCq	EUTH			
T5	35.23	28.08	NoCq	NoCq	NoCq	NoCq	NoCq	NoCq	NoCq	EUTH			
T6	31.8	29.80	NoCq	NoCq	NoCq	NoCq	NoCq	NoCq	NoCq	EUTH			
T7	29.81	29.40	NoCq	NoCq	NoCq	NoCq	NoCq	NoCq	NoCq	EUTH			
T8	32.35	27.90	NoCq	NoCq	NoCq	NoCq	NoCq	NoCq	NoCq	EUTH			
T9	30.56	26.66	NoCq	NoCq	NoCq	NoCq	NoCq	NoCq	NoCq	EUTH			
T10	34.03	36.00	NoCq	NoCq	NoCq	NoCq	NoCq	NoCq	NoCq	NoCq	NoCq	NoCq	NoCq
T11	34.76	35.49	NoCq	NoCq	NoCq	NoCq	NoCq	NoCq	NoCq	NoCq	NoCq	NoCq	NoCq
T12	34.34	34.49	NoCq	NoCq	NoCq	NoCq	NoCq	NoCq	NoCq	NoCq	NoCq	NoCq	NoCq
T13	32.43	NoCq	NoCq	NoCq	NoCq	NoCq	NoCq	NoCq	NoCq	NoCq	NoCq	NoCq	NoCq
T14	NoCq*	NoCq	NoCq	NoCq	NoCq	NoCq	NoCq	NoCq	NoCq	NoCq	NoCq	NoCq	NoCq
T15	38.44	36.41	NoCq	NoCq	NoCq	NoCq	NoCq	NoCq	NoCq	NoCq	NoCq	NoCq	NoCq
T16	NoCq	29.52	NoCq	NoCq	NoCq	NoCq	NoCq	NoCq	NoCq	NoCq	NoCq	NoCq	NoCq
T17	31.67	32.35	NoCq	NoCq	NoCq	NoCq	NoCq	NoCq	NoCq	LTF			
T18	28.49	27.01	NoCq	NoCq	NoCq	NoCq	NoCq	NoCq	NoCq	NoCq	NoCq	NoCq	NoCq
T19	34.25	30.32	NoCq	NoCq	NoCq	NoCq	NoCq	NoCq	NoCq	NoCq	NoCq	NoCq	NoCq
**Control**													
C1	25.89		NoCq	40.35	34.56	NoCq	33.49	27.22	ND	EUTH			
C2	31.03	29.84	NoCq	NoCq	NoCq	NoCq	NoCq	NoCq	NoCq	EUTH			
C3	33.29	38.45	31.71	41.55	39.61	NoCq	NoCq	34.39	35.61	34.79	ND	ND	ND

Table S7. Quantitation cycle (Cq) at which product is detected in DNA isolated from blood. EUTH indicates time of euthanasia. LTF = lost to follow-up. *Note animal T14 was negative by PCR at the 2 pre-treatment sample points but was previously positive (Cq of 29.56 in Jan 2017 (8 months before treatment) and was hemoculture positive in pre-treatment bleed (see Table S8).

Animal ID	Quad single	Quad pool	Heart single	Heart pool	Bicep single	Bicep pool	Sm intestine single	Lrg intestine single	Lrg intestine pool	Eso-phagus single	Eso-phagus pool	Tongue single	Tongue pool	Liver single	Liver pool	Fat single	Fat pool	Back muscle single	Back muscle pool	Brain single	Brain Pool	Spleen single	Total# tissue biopsies
Treated																							
T1	0/8	0/2	0/8	0/2	0/3	0/1		0/3	0/1	0/3	0/1			0/3	0/1	0/3	0/1			0/3	0/1		84
T2	0/8	0/2	0/8	0/2	0/3	0/1		0/3	0/1	0/3	0/1			0/3	0/1	0/3	0/1			0/3	0/1		84
T3	0/8	0/2	0/8	0/2	0/3	0/1		0/3	0/1	0/3	0/1			0/3	0/1	0/3	0/1			0/3	0/1		84
T4	0/8	0/2	0/13	0/2	0/8	0/1		0/3	0/1	0/3	0/1			0/3	0/1	0/8	0/1			0/3	0/1		99
T5	0/8	0/2	0/8	0/2	0/3	0/1		0/3	0/1	0/3	0/1			0/3	0/1	0/3	0/1			0/3	0/1		84
T6	0/8	0/2	0/8	0/2	0/3	0/1		0/3	0/1	0/3	0/1			0/3	0/1	0/3	0/1			0/3	0/1		84
T7	0/8	0/2	0/8	0/2	0/3	0/1		0/3	0/1	0/3	0/1			0/3	0/1	0/3	0/1			0/3	0/1		84
T8	0/8	0/2	0/8	0/2	0/3	0/1		0/3	0/1	0/3	0/1			0/3	0/1	0/3	0/1			0/3	0/1		84
T9	0/8	0/2	0/8	0/2	0/3	0/1		0/3	0/1	0/3	0/1			0/3	0/1	0/3	0/1			0/3	0/1		84
Control																							
C1	2/14	2/2	2/9	0/1	5/11	2/2	0/2	0/4	1/1	3/3				2/3	0/1	1/5	0/1	0/4	0/1			0/4	104
C2	0/14	0/8	0/13	0/7	0/9	0/6	0/5	0/11	0/3	0/10	0/1	0/10	0/1	0/10	0/1	0/10	0/1			0/9	0/2	0/3	254

Table S8. Detection of parasite DNA in tissue samples from treated and control (not treated) macaques. DNA isolated from individual specimens or pools of 5 specimens from different locations in the tissue/organ were screened by PCR for detection of *T. cruzi* DNA. Data indicate number of PCR+ samples/number of samples tested. Back muscle is the latissimus dorsi. Total specimens = the sum of individual and pooled specimens screened.

Data S1. (separate file). Pre-, during- and post-treatment clinical data for NHP.

Source Data image for S1a

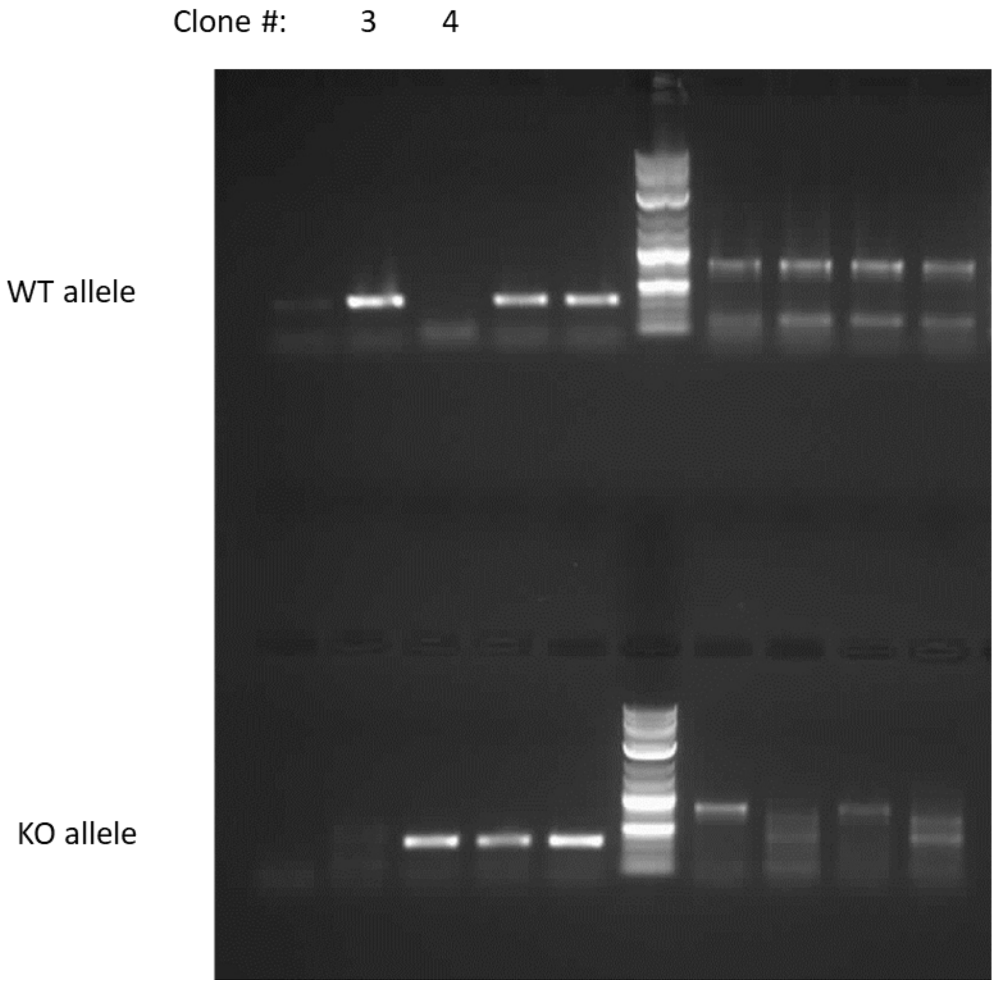

Supplement: Supplementary file 1 [file 41564_2022_1211_MOESM1_ESM.pdf]
