# Supplementary material for: Discovery of an orally active benzoxaborole prodrug effective in the treatment of Chagas disease in non-human primates
Source: Nat Microbiol. 2022 Sep 5;7(10):1536–46. doi: 10.1038/s41564-022-01211-y (PMC9519446; doi:10.1038/s41564-022-01211-y)
Supplement: Supplementary file 2 [file 41564_2022_1211_MOESM2_ESM.pdf]

nature portfolio

Corresponding author(s): Rick L. Tarleton

Last updated by author(s): Jul 6, 2022

Reporting Summary

Nature Portfolio wishes to improve the reproducibility of the work that we publish. This form provides structure for consistency and transparency in reporting. For further information on Nature Portfolio policies, see our Editorial Policies and the Editorial Policy Checklist.

Statistics

For all statistical analyses, confirm that the following items are present in the figure legend, table legend, main text, or Methods section.

n/a	Confirmed	
☐	☒	The exact sample size (*n*) for each experimental group/condition, given as a discrete number and unit of measurement
☐	☒	A statement on whether measurements were taken from distinct samples or whether the same sample was measured repeatedly
☐	☒	The statistical test(s) used AND whether they are one- or two-sided<br>*Only common tests should be described solely by name; describe more complex techniques in the Methods section.*
☒	☐	A description of all covariates tested
☒	☐	A description of any assumptions or corrections, such as tests of normality and adjustment for multiple comparisons
☐	☒	A full description of the statistical parameters including central tendency (e.g. means) or other basic estimates (e.g. regression coefficient) AND variation (e.g. standard deviation) or associated estimates of uncertainty (e.g. confidence intervals)
☐	☒	For null hypothesis testing, the test statistic (e.g. *F*, *t*, *r*) with confidence intervals, effect sizes, degrees of freedom and *P* value noted<br>*Give P values as exact values whenever suitable.*
☒	☐	For Bayesian analysis, information on the choice of priors and Markov chain Monte Carlo settings
☒	☐	For hierarchical and complex designs, identification of the appropriate level for tests and full reporting of outcomes
☒	☐	Estimates of effect sizes (e.g. Cohen's *d*, Pearson's *r*), indicating how they were calculated

Our web collection on statistics for biologists contains articles on many of the points above.

Software and code

Policy information about availability of computer code

Data collection	Living Imaging Software v4.0 (PerkinElmer)<br>Maestro 2.10 (CRi)<br>Biorad CFX manager software v3.1 (Bio-Rad)<br>Gene5 v 2.0 (BioTek)<br>ImageLab Touch 2.4.03 (Bio-Rad)<br>HiSAT software package v0.1.6 (http://www.ccb.jhu.edu/software/hisat/index.shtml)<br>HTseq v0.6.1 (https://htseq.readthedocs.io/en/master/)<br>TargetLynx v4.1 (Waters)
Data analysis	GraphPad Prism v9.4.0 (GraphPad Software)

For manuscripts utilizing custom algorithms or software that are central to the research but not yet described in published literature, software must be made available to editors and reviewers. We strongly encourage code deposition in a community repository (e.g. GitHub). See the Nature Portfolio guidelines for submitting code & software for further information.

Data

Policy information about availability of data

All manuscripts must include a data availability statement. This statement should provide the following information, where applicable:

- Accession codes, unique identifiers, or web links for publicly available datasets
- A description of any restrictions on data availability
- For clinical datasets or third party data, please ensure that the statement adheres to our policy

_With the exception of mRNASeq data (present in the NCBI 3 Sequence Read Archive (SRA; http://www.ncbi.nlm.nih.gov/sra/) under accession numbers are: SRX13363525 - SRX13363532), all data are available in the main text or the supplementary materials.
_CL Brener genome (TritrypDB release-33); (https://tritrypdb.org/tritrypdb/app)
_African green monkey genome (The DNA Data Bank of Japan (DDBJ, http://www.ddbj.nig.ac.jp); accession number: DRA002256)

Field-specific reporting

Please select the one below that is the best fit for your research. If you are not sure, read the appropriate sections before making your selection.

☒ Life sciences ☐ Behavioural & social sciences ☐ Ecological, evolutionary & environmental sciences

For a reference copy of the document with all sections, see nature.com/documents/nr-reporting-summary-flat.pdf

Life sciences study design

All studies must disclose on these points even when the disclosure is negative.

Sample size	For in vivo testing of compound efficacy in mice, the sample size was based upon previous experiments that allowed discrimination between effective and ineffective treatments (Bustamante JM, et al., New, combined, and reduced dosing treatment protocols cure Trypanosoma cruzi infection in mice. J Infect Dis. 2014 Jan 1;209(1):150-62. doi: 10.1093/infdis/jit420. Epub 2013 Aug 14. PMID: 23945371). For the trial in NHP, a power analysis (one-sided 95% Confidence Intervals) determined that in order to have a >85% confidence of concluding that a compound is 100% effective, we would need 19 animals in the treatment group. To reduce animal numbers while evaluating the test compound on its own (without comparison to the other therapies), and also providing sufficient power to the analysis, we used 19 animals in a single treatment group (thus no blinding or randomization was required).
Data exclusions	No data were excluded from analysis.
Replication	Experimental findings of the mouse studies were reproducible in independent experiments performed at least twice. All in vitro parasite proliferation assays were repeated at least one time with similar results. PCR and Western blot assays depicted as representative microphotographs in Fig 2b were repeated three and one time respectively with similar results. The quantitative liquid chromatography tandem mass spectrometry assay described in Fig. 2d was performed once. Due to cost and complexity, the NHP trial was performed once.
Randomization	Mice and other samples were randomly allocated in the experimental groups.
Blinding	Investigators were blinded to group allocation during collection data.

Reporting for specific materials, systems and methods

We require information from authors about some types of materials, experimental systems and methods used in many studies. Here, indicate whether each material, system or method listed is relevant to your study. If you are not sure if a list item applies to your research, read the appropriate section before selecting a response.

Materials & experimental systems

n/a	Involved in the study	
☐	☒	Antibodies
☐	☒	Eukaryotic cell lines
☒	☐	Palaeontology and archaeology
☐	☒	Animals and other organisms
☒	☐	Human research participants
☒	☐	Clinical data
☒	☐	Dual use research of concern

Methods

n/a	Involved in the study	
☒	☐	ChIP-seq
☒	☐	Flow cytometry
☒	☐	MRI-based neuroimaging

Antibodies

Antibodies used	_Macaque antibody binding to individual beads in the Multiplex assays was detected with donkey anti-human IgG (H+L) conjugated

Antibodies used	to phycoerythrin (cat no. 709-116-149, Jackson ImmunoResearch); 1:200 dilution.<br>_IRDye 800CW Donkey anti-Rabbit IgG (cat no. 926-32213; Li-COR); 1:10,000 dilution.<br>_B-tubulin antibody (cat no. 2146S; Cell Signaling); 1:1000 dilution.<br>_TcCBP-specific antibody (gift of Dr. Juan José Cazzulo at Instituto de Investigaciones Biotecnológicas); 1:500 dilution.
Validation	Antibodies used in this study have successfully been validated by the manufacturer and used in previous works involving human and macaque samples.<br><br>Cooley et. al. PLoS Negl Trop Dis. 2008 Oct 8;2(10):e316. doi: 10.1371/journal.pntd.0000316.<br>Padilla et. al. PLoS Negl Trop Dis. 2021 Mar 31;15(3):e0009141. doi: 10.1371/journal.pntd.0009141. eCollection 2021.

Eukaryotic cell lines

Policy information about cell lines

Cell line source(s)	Vero cells were obtained from the American Type Culture Collection (Manassas, VA).<br>Wild type and genetically modified lines of Trypanosoma cruzi are maintained in our laboratory.
Authentication	Vero cell line was not authenticated.<br>Trypanosoma cruzi discrete typing units (DTU) of the parasite lines in our laboratory are identified by PCR.
Mycoplasma contamination	Cell lines and parasites used in the experiments tested negative for Mycoplasma contamination.
Commonly misidentified lines<br>(See ICLAC register)	No misidentified cell lines were used in this study

Animals and other organisms

Policy information about studies involving animals; ARRIVE guidelines recommended for reporting animal research

Laboratory animals	Male and female mice, 6-9 weeks old, from the strains C57BL/6J; B6.129S7-Ifngtm1Ts/J (IFN-gamma deficient) and SKH-1 "hairless" mice backcrossed to C57BL/6 were used in this study. Mice were maintained in the University of Georgia Animal Facility under specific pathogen–free conditions at 22°C, 50% humidity and in a 12:12 hs light:dark cycle.<br>Male and female Rhesus Macaques (Macaca mulatta), 3-23 years old were used in this study.
Wild animals	This study did not involve wild animals.
Field-collected samples	This study did not involve samples collected in the field.
Ethics oversight	The mouse experiments were carried out in strict accordance with the Public Health Service Policy on Humane Care and Use of Laboratory Animals and Association for Assessment and Accreditation of Laboratory Animal Care accreditation guidelines. The protocol was approved by the University of Georgia Institutional Animal Care and Use Committee<br>All protocols used with NHPs were approved by the MD Anderson Cancer Center's IACUC, and followed the NIH standards established by the Guide for the Care and Use of Laboratory Animals.

Note that full information on the approval of the study protocol must also be provided in the manuscript.
